# The Impact of Graphite Oxide Nanocomposites on the Antibacterial Activity of Serum

**DOI:** 10.3390/ijms22147386

**Published:** 2021-07-09

**Authors:** Katarzyna Dorota Morka, Maciej Wernecki, Anna Kędziora, Marta Książczyk, Bartłomiej Dudek, Yuriy Gerasymchuk, Anna Lukowiak, Jarosław Bystroń, Gabriela Bugla-Płoskońska

**Affiliations:** 1 Department of Food Hygiene and Consumer Health Protection, Faculty of Veterinary Medicine, University of Environmental and Life Sciences, C. K. Norwida 31, 50-375 Wrocław, Poland; jaroslaw.bystron@upwr.edu.pl; 2 Department of Microbiology, Faculty of Biological Sciences, University of Wroclaw, S. Przybyszewskiego 63/77, 51-148 Wroclaw, Poland; maciej.wernecki@uwr.edu.pl (M.W.); anna.kedziora@uwr.edu.pl (A.K.); marta.ksiazczyk@uwr.edu.pl (M.K.); bartlomiej.dudek@uwr.edu.pl (B.D.); 3 Institute of Low Temperature and Structure Research, Polish Academy of Sciences, ul. Okolna 2, 50-422 Wrocław, Poland; y.gerasymchuk@intibs.pl (Y.G.); a.lukowiak@intibs.pl (A.L.)

**Keywords:** GO nanocomposites, photoactivity, bactericidal action of serum, *E. coli*

## Abstract

Nanoparticles can interact with the complement system and modulate the inflammatory response. The effect of these interactions on the complement activity strongly depends on physicochemical properties of nanoparticles. The interactions of silver nanoparticles with serum proteins (particularly with the complement system components) have the potential to significantly affect the antibacterial activity of serum, with serious implications for human health. The aim of the study was to assess the influence of graphite oxide (GO) nanocomposites (GO, GO-PcZr(Lys)_2_-Ag, GO-Ag, GO-PcZr(Lys)_2_) on the antibacterial activity of normal human serum (NHS), serum activity against bacteria isolated from alveoli treated with nanocomposites, and nanocomposite sensitivity of bacteria exposed to serum in vitro (using normal human serum). Additionally, the in vivo cytotoxic effect of the GO compounds was determined with application of a *Galleria mellonella* larvae model. GO-PcZr(Lys)_2_, without IR irradiation enhance the antimicrobial efficacy of the human serum. IR irradiation enhances bactericidal activity of serum in the case of the GO-PcZr(Lys)_2_-Ag sample. Bacteria exposed to nanocomposites become more sensitive to the action of serum. Bacteria exposed to serum become more sensitive to the GO-Ag sample. None of the tested GO nanocomposites displayed a cytotoxicity towards larvae.

## 1. Introduction

Recently, there was increasing research on the interactions of graphite oxide (GO) as well as various graphene products obtained from graphite oxide with biological objects [1]. Various mechanisms of the influence of these materials on living cells, both eukaryotic and prokaryotic, are discussed. Materials based on graphene are also tested in terms of their application in diagnostics [2,3], where both carbon materials, for example, quantum dots [4,5], and composite systems are developed [6]. One of the potential directions of application of these materials is their use in photodynamic therapies including antibacterial activity, which is particularly interesting as an alternative to antibiotic therapy. Graphene and graphene products have more interesting and favorable physicochemical properties compared to that of graphite oxide, but their disadvantage is that they do not contain active groups, which can be chemically modified, and their introduction into the graphene structure is very complicated and is associated with disturbance of the native structure and loss of their properties. In terms of chemical modification, graphite oxide is more interesting because it has many active groups containing oxygen on its surface which can be chemically modified without significantly disturbing its structure.

As a component of composite materials, especially photoactive, graphite oxide plays a triple role. On the one hand, it can act as an antenna for photons, with the subsequent transfer of energy obtained from the light to photosensitizer particles or luminescent nanoparticles, and on the other hand, it allows for enhancement of the antibacterial effect of silver (both in ionic and nanoparticle forms) [7,8,9,10,11]. A photosensitizer—a complex of zirconium (IV) phthalocyanine—is herein proposed as a photoactive component bonded to GO flakes. Its presence ensures generation of singlet oxygen molecules after near infrared light exposure, which may cause death of bacterial cells. To strengthen the bactericidal effect, silver nanoparticles can be introduced to the system as well.

More than 700 prokaryotic species in the human oral cavity, belonging to 13 separate phyla, are noted in the literature. Due to the specific environmental conditions, the species diversity within the infected root canal is usually limited, up to 10–30 species per canal [12]. Aerobic (such as streptococci, staphylococci, enterococci, *Enterobacteriaceae*), anaerobic (such as *Fusobacterium*, *Prevotella* and *Porphyromonas* spp.) and microaerophiles (such as *Lactobacillus***,** *Actinomyces,* and *Propionibacteria* spp.) are the bacteria most frequently isolated from the root canal [13].

Besides the previous studies, where the antibacterial activity of GO nanocomposites was determined, we decided to check the impact of reduced GO (rGO) nanocomposites on the antibacterial efficacy of serum after recognition that nanomaterials introduced into the body tend to influence the complement system and can interact with different components of the serum, including immunologically significant complement proteins [14,15]. A proteomic study [16] demonstrated that some serum proteins can bind to nanoparticles. The total amount and type of proteins bound to nanoparticles depend on the physicochemical properties of the nanoparticles. However, the implications of the binding of serum proteins to silver nanoparticles regarding the complement activation and immune response against bacteria were not fully investigated.

Serum contains crucial elements of immunity as part of the host defense system, such as immunoglobulin or the complement system. The complement system (35–40 proteins in the blood plasma) plays a fundamental role in mediating and enhancing humoral immunity. Under normal conditions, activation of any of the three complement pathways—the classical, alternative, or lectin—results in formation of the membrane attack complex (MAC). When this activation occurs in the presence of antigens (e.g., outer membrane structures of bacteria), the generated MAC may form transmembrane pores in the phospholipid bilayer of the targeted bacteria cell, causing complement-mediated cytolysis [17].

Because mammalian models of infection have numerous disadvantages such as high cost, ethical constraints, and specialized equipment and laboratories, a promising and reliable alternative method to investigate the in vivo activity of antimicrobial agents is invertebrate models, such as *Galleria mellonella* (greater wax moth) larvae [18,19]. The larvae were widely used as an infection model to study a large range of pathogens [20,21,22,23,24]. The reason why *G. mellonella* larvae have application in in vivo pathogenicity, toxicity, and antimicrobial activity testing is the high degree of similarity of the larval immune system to the innate immune system of mammals, including humans [23]. Previous studies showed a strong positive correlation of virulence of different pathogens between mouse infection systems and *G. mellonella* [20,21,22,23,24]. In the present study we applied tested nanocomposites to *G. mellonella* larvae as an alternative model to examine the cytotoxicity of designed nanocomposites and predict their suitability for clinical use in humans. The aim of the study was to assess the influence of GO nanocomposites on the antibacterial activity of normal human serum (NHS), serum activity against bacteria treated with nanocomposites, and nanocomposite sensitivity of bacteria exposed to serum in vitro and in vivo (using the *G. mellonella* model). The model of research using GO with serum and *G. mellonella* larva interaction was not previously presented in the literature. The following materials were used for testing: GO, GO with silver nanoparticles (GO-Ag), GO with attached molecules of bis(lysinato)zirconium(IV) phthalocyanine complex (GO-PcZr(Lys)_2_), and the latter with silver nanoparticles (GO-PcZr(Lys)_2_-Ag).

## 2. Results

### 2.1. Survival of E. coli 6.2E in NHS, iNHS (Inactivated Serum) and Nanocomposites (GO, GO-PcZr(Lys)_2_-Ag, GO-Ag, GO-PcZr(Lys)_2_) with and without IR Irradiation

**Figure 1:**
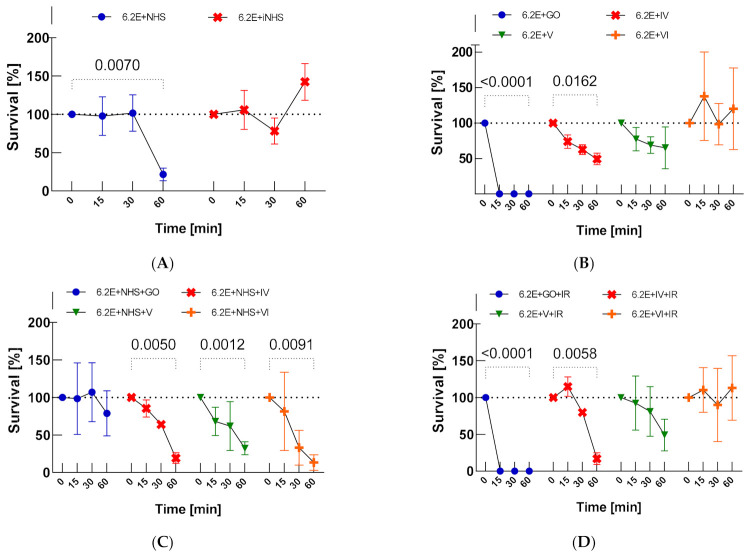
Bacterial survival in: NHS and iNHS (**A**), nanocomposites GO, IV: GO-PcZr(Lys)_2_-Ag, V: GO-Ag, VI: GO-PcZr(Lys)_2_ without IR irradiation (**B**), nanocomposites GO, IV: GO-PcZr(Lys)_2_-Ag, V: GO-Ag, VI: GO-PcZr(Lys)_2_ with IR irradiation (**D**) and mixed NHS with nanocomposites (**C**). Graphs represent the percentage of live bacteria in the sample relative to the average number at time zero. Standard deviation values are presented with error bars, Dunnett’s test *p*-values for within group T0–T60 pairs are shown above graph when equal or lower than 0.05. Mixed-model ANOVA results are presented in Table A3 in Appendix D. Corresponding results obtained for strain *E. coli J53* are presented in Figure A5 in Appendix C.

First, the efficacy of 25% NHS against *E. coli* 6.2E isolated from alveoli was determined; this constituted a control for further experiments. The results showed that NHS shows bactericidal activity against the tested *E. coli* 6.2E strain during 60 min. *E. coli* 6.2E maintained the ability to survive in 25% NHS during the tested time (at T60, bacterial CFU/mL was equal to 1.39 × 10^6^ (as illustrated in Table A1), which is 22% of the CFU/mL at T0, when the bacterial survival rate is 100%, *p* = 0.0070), while in iNHS the strain multiplied (142% survival, *p* > 0.05, Figure 1A). The addition of GO-PcZr(Lys)_2_-Ag and GO-PcZr(Lys)_2_ to the environment of NHS and bacterial cells did not significantly enhanced the decrease in bacterial count compared to NHS (19% (*p* = 0.0050) and 13% (*p* = 0.0091) survival, respectively), while in iNHS and GO-PcZr(Lys)_2_-Ag the strain CFU/mL decreased insignificantly after 60 min (CFU/mL at T0 equal to 5.82 × 10^6^ decreased to 4.23 × 10^6^). One hour is not enough for nanocomposites GO-PcZr(Lys)_2_-Ag and GO-PcZr(Lys)_2_ simultaneously added to NHS and *E. coli* 6.2.E to block or stimulate the complement activity (as illustrated in Figure 1C).

Incubation of *E. coli* 6.2E with GO-PcZr(Lys)_2_-Ag (*p* = 0.0162), GO-Ag (*p* > 0.05), and GO (*p* < 0.0001) showed reduction of the number of bacteria (as illustrated in Figure 1B). The bactericidal effect was enhanced by light exposure (broadband light in the red and near-infrared region, IR) only in the case of nanocomposite GO-PcZr(Lys)_2_-Ag (as illustrated in Figure 1D, *p* = 0.0058).

### 2.2. The Influence of Nanocomposites (GO, GO-PcZr(Lys)_2_-Ag, GO-Ag, GO-PcZr(Lys)_2_) on the Bacterial Serum Survival

**Figure 2:**
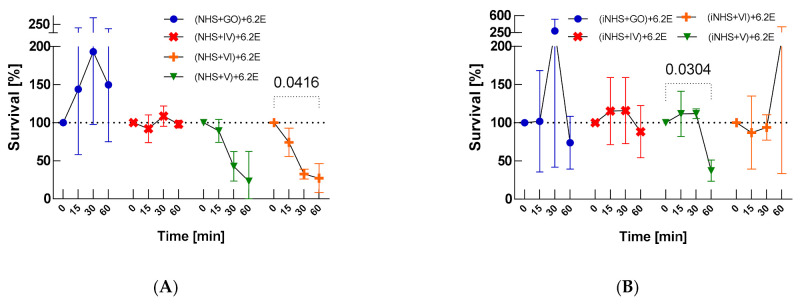

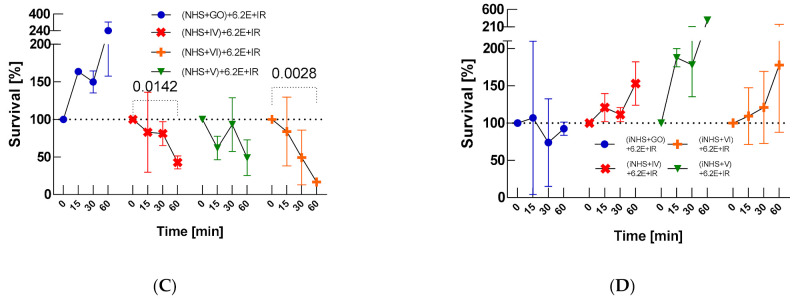
Bactericidal effectiveness of NHS exposed to nanocomposites (GO, IV: GO-PcZr(Lys)_2_-Ag, V: GO-Ag, VI: GO-PcZr(Lys)_2_) before (**A**) and after IR irradiation (**C**), the control test in iNHS (**B**,**D**). Graphs represent percentage of live bacteria in sample relative to average number at time zero. Standard deviation values are presented with error bars, Dunnett’s test *p*-values for within group T0–T60 pairs are shown above graph when equal or lower than 0.05. Mixed-model ANOVA results are presented in Table A3. Corresponding results obtained for strain *E. coli J53* are presented in Figure A6.

The nanocomposites influenced the bacterial serum survival. There was a slight decrease in CFU/mL of *E. coli* 6.2E in GO-PcZr(Lys)_2_-Ag-exposed NHS from 6.62 × 10^6^ at T0 to 6.5 × 10^6^ at T60, which is 98% of the T0 value (*p* > 0.05, as illustrated in Figure 2A). Similarly, this strain displayed high-level resistance to GO-treated serum (150% survival, *p* > 0.05) (as illustrated in Figure 2A). In contrast, there was 22% survival of bacteria in the active NHS (as illustrated in Figure 1A) and survival of 19% of bacteria after simultaneous mixing with NHS and GO-PcZr(Lys)_2_-Ag (as illustrated in Figure 1C). However, the GO-Ag and GO-PcZr(Lys)_2_ nanocomposites led to a decrease of bacterial survival in serum (23% (*p* > 0.05) and 27% (*p* = 0.0416) survival, respectively, as illustrated in Figure 2A) and GO-Ag maintained its ability to reduce bacteria at a similar level as observed in GO-Ag-treated iNHS (survival decreased to 37%, *p* = 0.0304, as illustrated in Figure 2B). The bactericidal effect was mainly influenced by the serum, and the GO-Ag nanocomposite played a supporting role in this phenomenon. On the other hand, a slight decrease in CFU/mL from T0 4.59 × 10^6^ to 3.71 × 10^6^ at T60 was recorded for *E. coli* 6.2E in GO-PcZr(Lys)_2_-Ag-treated inactivated serum (88% survival, *p* > 0.05, as illustrated in Figure 2B). A similar decrease was also observed for GO-treated iNHS; the strain exhibited 74% survival *p* > 0.05.

**Figure 3:**
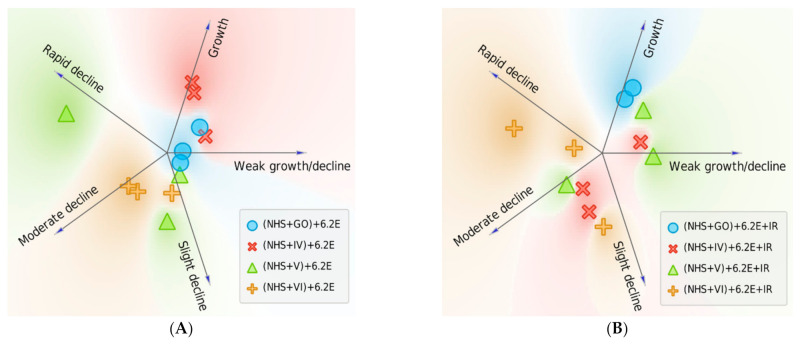
Survival of *E. coli* 6.2E was altered when bacteria were exposed to IR irradiated NHS with nanocomposites. Each replicate is represented with an individual sign and placed according to the probability of belonging to a particular cluster (numerical data shown in Table A3). Survival was decreased with the nanocomposite GO-PcZr(Lys)_2_-Ag (note the shift of the red Xs from cluster ‘Growth’ (**A**) closer to cluster ‘Moderate decline’ (**B**)).

Significant enhancement (*p* = 0.0117, between T60 survival values from tests (NHS+IV)+6.2E withut IR vs. (NHS+IV)+6.2E+IR) of the bactericidal effect was observed in GO-PcZr(Lys)_2_-Ag-treated NHS, subsequently IR-exposed (decrease of CFU/mL from 4.37 × 10^6^ to 1.88×10^6^ after 60 min of incubation) (as illustrated in Figure 2C). Thus, the previously observed insignificant reduced bactericidal ability shown by GO-PcZr(Lys)_2_-Ag-treated NHS (as illustrated in Figure 2A) was intensified by IR light (*p* = 0.0142). However, in the case of iNHS exposed to GO-PcZr(Lys)_2_-Ag nanocomposite ((iNHS + GO-PcZr(Lys)_2_-Ag) + *E. coli* 6.2E + IR) the IR did not maintain the GO-PcZr(Lys)_2_-Ag-mediated ability to reduce the number of bacteria as was the case in the experiment without IR irradiation (as illustrated in Figure 2B). The reactions without GO-PcZr(Lys)_2_-Ag nanocomposite excluded the possibility that IR light induced the local heating of the serum (data not shown). The role of IR irradiation in reducing the number of bacteria was not clearly observed in GO, GO-Ag, and GO-PcZr(Lys)_2_-exposed NHS (240%, 50% and 17% survival, respectively, as illustrated in Figure 2C), because without IR the strain displayed similar susceptibility (150%, 23% and 27% after 60 min of incubation, as illustrated in Figure 2A). The summarized clustering analysis is presented in Figure 3.

### 2.3. Serum Activity against Bacteria Treated with Nanocomposites and Nanocomposite Sensitivity of Bacteria Exposed to Serum

**Figure 4:**
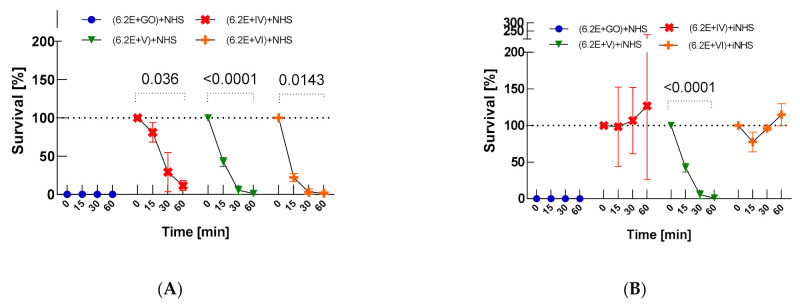
Serum sensitivity of *E. coli* 6.2E exposed to nanocomposites (GO, IV: GO-PcZr(Lys)_2_-Ag, V: GO-Ag, VI: GO-PcZr(Lys)_2_) (**A**) and control test in iNHS (**B**). Graphs represent percentage of live bacteria in sample relative to average number at time zero. Standard deviation values are presented with error bars, Dunnett’s test *p*-values for within group T0–T60 pairs are shown above graph when equal or lower than 0.05. Mixed-model ANOVA results are presented in Table A3. Corresponding results obtained for strain *E. coli J53* are presented in Figure A7.

The most bactericidal effect and also the fastest reduction rate of the bacterial CFU/mL were observed after nanocomposite treatment, which may suggest that the nanocomposites make bacteria more susceptible to the complement action. Only 12% (*p* = 0.0036), 1% (*p* < 0.0001), and 1% (*p* = 0.0143) of bacteria exposed to GO-PcZr(Lys)_2_-Ag, GO-Ag, and GO-PcZr(Lys)_2_ nanocomposites, respectively, survived the human serum activity. *E. coli* 6.2E showed high susceptibility to GO action with a survival rate of 0% after 30-min incubation before the serum treatment (as illustrated in Figure 4A).

**Figure 5:**
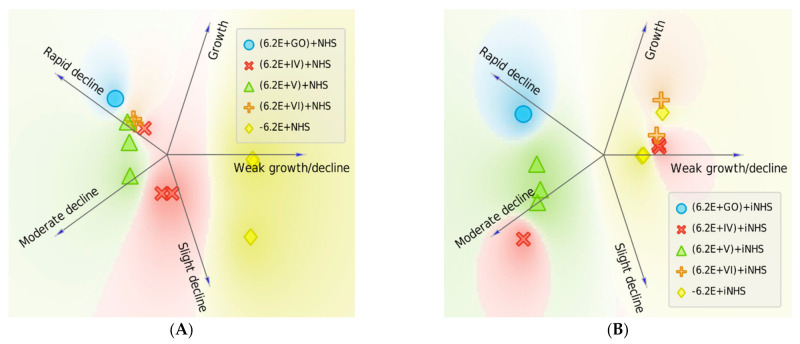
Survival of *E. coli* 6.2E was reduced when bacteria were exposed to nanocomposites followed by NHS treatment. Each replicate is represented with an individual sign and placed according to the probability of belonging to a particular cluster (numerical data shown in Table A3). Effect is notable with nanocomposites IV: GO-PcZr(Lys)_2_-Ag, V: GO-Ag, VI: GO-PcZr(Lys)_2_ (shift to the ‘decline’ clusters when compared to baseline 6.2E + NHS experiment, (**A**). Effect of nanocomposite-induced sensitization is maintained in iNHS only for nanocomposite V: GO-Ag, for which all replicates are clustered as ‘moderate decline’ (**B**).

Serum survival of nanocomposites-treated *E. coli* 6.2E was many times lower compared to bacteria not exposed to GO, GO-PcZr(Lys)_2_-Ag, GO-Ag, and GO-PcZr(Lys)_2_ action, which may suggest that the interaction of bacteria with nanocomposites facilitates the NHS activity (as illustrated in Figure 1C, Figure 4A). On the other hand, the changes caused by GO-PcZr(Lys)_2_-Ag and GO-PcZr(Lys)_2_ nanocomposites were not so severe, so bacterial cells were able to multiply again in iNHS. GO-PcZr(Lys)_2_-Ag and GO-PcZr(Lys)_2_-exposed *E. coli* 6.2E exhibited 127% and 115% (*p* > 0.05) survival in iNHS, respectively, after 60-min incubation (as illustrated in Figure 4B). The GO-Ag-treated strain displayed a low level of survival in iNHS (1% after 60 min incubation, *p* = 0.0001), while GO exposure led to total death of bacteria. The summarized clustering analysis is presented in Figure 5.

**Figure 6:**
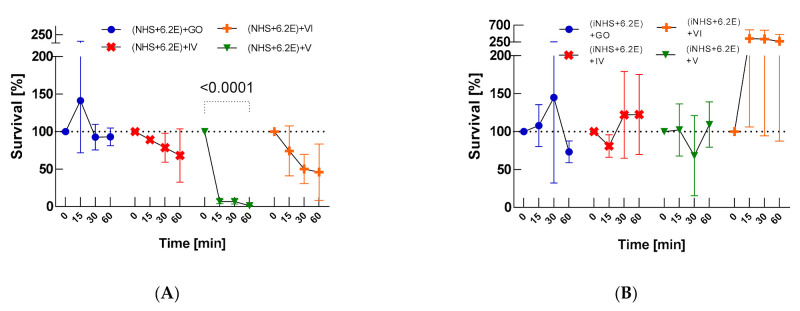
Sensitivity of *E. coli* 6.2E to nanocomposites (GO, IV: GO-PcZr(Lys)_2_-Ag, V: GO-Ag, VI: GO-PcZr(Lys)_2_) after exposure to serum (**A**) and control test in iNHS (**B**). Graphs represent percentage of live bacteria in sample relative to the average number at time zero. Standard deviation values are presented with error bars, Dunnett’s test *p*-values for within group T0–T60 pairs are shown above graph when equal or lower than 0.05. Mixed-model ANOVA results are presented in Table A3. Corresponding results obtained for strain *E. coli J53* are presented in Figure A8.

Treatment of bacteria with NHS for 30 min prior to the action of nanocomposites did not lead to the death of the bacterial population. The strain exhibited 68%, 46%, and 93% survival for GO-PcZr(Lys)_2_-Ag, GO-PcZr(Lys)_2,_ and GO, respectively, after 60 min of the experiment (*p* > 0.05). Only GO-Ag nanocomposite statistically significantly reduced the survival of NHS-exposed bacteria to 1% (*p* < 0.0001) (as illustrated in Figure 6A).

In contrast, there were 19%, 13%, and 79% of CFU/mL of surviving bacteria when no NHS treatment was applied to bacteria (as illustrated in Figure 1C), which may suggest that such an interaction of bacteria with NHS blocks the action of the compound GO-PcZr(Lys)_2_-Ag, GO-PcZr(Lys)_2_ and GO on bacteria.

**Figure 7:**
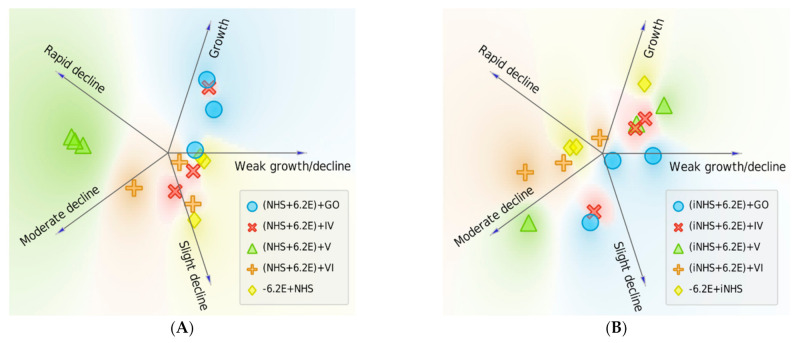
Survival of *E. coli* 6.2E was changed when bacteria were treated with nanocomposites exposed to NHS. Each experiment is represented with an individual sign and placed according to probability of belonging to a particular cluster (numerical data shown in Table A3). Bactericidal effect was enhanced in the experiment with nanocomposite V: GO-Ag, where NHS pretreatment caused a shift towards ‘Moderate decline’ and ‘Rapid decline’ clusters (**A**). The opposite effect was observed for GO, where contact with NHS reduced its effectiveness. When iNHS was applied, no nanocomposite maintained rapid bactericidal action (**B**).

Only GO reduced the bacterial population exposed to inactivated NHS by up to 73% (as illustrated in Figure 6B, *p* > 0.05). This iNHS-exposed strain also exhibited 109%, 122%, and 268% survival after 60 min of incubation in GO-PcZr(Lys)_2_, GO-PcZr(Lys)_2_-Ag, and GO-Ag nanocomposites, respectively (*p* > 0.05) (as illustrated in Figure 6B). The summarized clustering analysis is presented in Figure 7.

### 2.4. Galleria mellonella—In Vivo Cytotoxicity Tests

**Figure 8:**
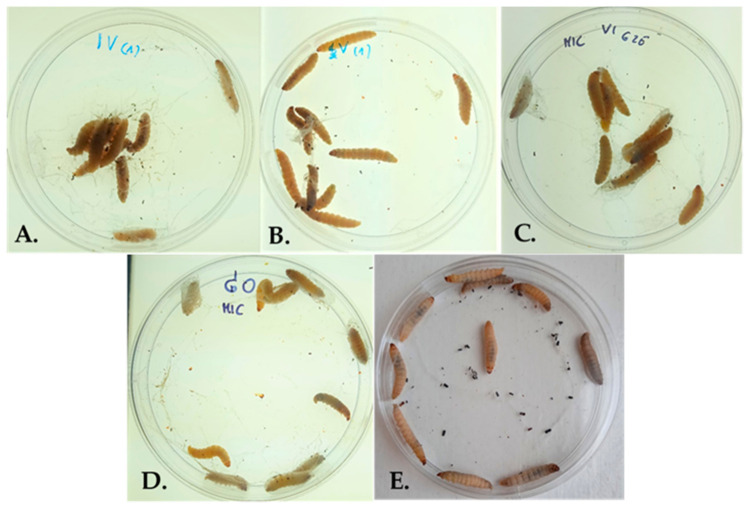
*G. mellonella* larvae after injection of following graphite nanocomposites in MIC concentration: GO-PcZr(Lys)_2_-Ag (**A**); GO-Ag (**B**); GO-PcZr(Lys)_2_ (**C**); GO (**D**); PBS control–Larvae (*n* = 10) injected with sterile PBS buffer (**E**).

In vivo cytotoxicity tests were conducted at least three times for each graphite nanocomposite. After separate application of graphite nanocomposites into the hemocoel of larvae (*n* = 10), the medium percentage survival of larvae was in the range of about 90–100% (as illustrated in Table 1). Nanocomposites in previously determined MIC concentrations exhibited no cytotoxic effect for *G. mellonella* larvae (as illustrated in Figure 8).

## 3. Discussion

The choice of phthalocyanines as photosensitizers in composite materials was dictated by the fact that, first of all, as a group of macrocyclic compounds, they were already used in traditional photodynamic therapy (e.g., for cancer treatment) as well as in antibacterial photodynamic therapy [25,26,27] due to high absorption coefficients and absorption and emission bands in the “biological window” range. The biological window is the range of the light spectrum in which it penetrates deeply (up to 2 cm) into the tissues of a living organism without being absorbed by biological substances and corresponds to the wavelength range of 600–900 nm. Moreover, the generation of reactive oxygen species under the influence of light from this range was proven for phthalocyanines, which is an additional advantage related to their action in photodynamic therapy. Phthalocyanine compounds might have an advantage over typical antiseptics (hypochlorite, chlorhexidine, or coumarins), which are unstable in the long term or toxic to the body when washed out by body fluids, and antibiotics, which also have low stability and at low concentrations lead to bacterial adaptation and formation of resistant strains rather than to the desired antiseptic effect [28,29].

Husain et al. [30] reported that a small fraction of nanomaterials can translocate from lungs to blood and can activate C3 protein (one crucial protein of the complement system). Long-term accumulation of nanoparticles can lead to chronic airway inflammation, where the complement system can be involved [31]. Keiser et al. [32] showed that in someone exposed to nano-TiO_2_ and nano-SiO_2_ at concentrations up to 243 μg/mL for 48 h, neither the gastrointestinal cells nor the immune system cells were significantly affected. However, when exposed to silver nanoparticles, several cell parameters were affected, but far less than by silver ions used as a control.

Hunag et al. [33] in 2016 described a study in which they investigated the blood biological effect of silver nanoparticles with two different surface coatings on serum immunity: polyvinyl pyrrolidone and polyvinyl pyrrolidone-citrate. They reported that those materials did not show any effect on complement activation at the concentration range from about 1 to 40 µg/mL. Yu et al. [31] found C3 as a protein bound on glycopolymer-grafted nanoparticles, which can modulate and amplify the complement system. Sladowski [34] and Moghimi et al. [35] concluded that silver nanoparticles with different physicochemical properties are involved only in the alternative pathway of complement activation, and nanoparticles with size 35 nm are better activators of complement than 25 nm and 10 nm. However, Fornaguera et al. [36] found no correlation between nanoparticle size and activation of the complement system.

There is a huge gap in the literature covering the influence of graphene nanocomposites on the nonspecific immunity and their antibacterial efficacy. Despite poor biosolubility and biocompatibility and induction of cell death, graphene oxide was considered as a promising vaccine carrier and adjuvant in activating cellular and humoral immunity [37]. Zhang et al. [38] confirmed the significant increase of host-immunity-related CD8^+^ T cells (cytotoxic T lymphocytes) and proinflammatory cytokines, including IFN-γ and TNF-α, after photo-activated antitumor activity with graphene quantum-dot-mediated photodynamic therapy. Cao et al. [37] stated that the surface modifications of graphene oxide and their functionalization are crucial for individual applications taking into consideration the biological interaction. The differences in interaction of graphite nanocomposites with human serum were noted by us. Final conclusions strongly depend on the physicochemical properties of nanomaterials. We found that pure GO or graphene oxide doped with silver and phthalocyanines complex (GO-PcZr(Lys)_2_-Ag) had a completely different influence on serum compounds and their antibacterial efficacy than GO-PcZr(Lys)_2_ or GO-Ag. The crucial points are the external factors such as IR irradiation, which we also observed, and it does not depend on the chemical composition of the sample. It is worth underlining that the obtained results also depend on the point of immunological response. The contact of an antigen (bacterial) with human serum occurs with the existence of other complement components. Antibacterial activity of human serum may decrease after contact with pure GO and GO-PcZr(Lys)_2_-Ag but increase after exposure to GO-PcZr(Lys)_2_ or GO with silver nanoparticles (with Ag diameter about 10 nm) added to these samples, enhancing antibacterial efficacy of graphite nanocompounds. As we previously noted, the response of bacterial cells to silver nanoparticles depends on the physicochemical properties of the nanoformulations (such as size, shape, charge, surface area, compounds, etc.) and individual features of bacterial strains (such as structural compounds and metabolism), and the incorporation of silver into industrial products should be considered to create a separate agent with a potentially different mode of antibacterial action [39].

Survival of *E. coli* 6.2E was tested in different environments, i.e., in 25% NHS, 25% iNHS, nanocomposites (GO, GO-PcZr(Lys)_2_-Ag, GO-Ag or GO-PcZr(Lys)_2_) with or without IR irradiation as a control being a reference for further reactions. The control experiment was also simultaneously mixed bacteria with nanocomposites and serum. These results showed that almost all (except for GO-PcZr(Lys)_2_ and GO-Ag, *p* > 0.05) of the mentioned environments had a reducing effect on the number of bacteria. *E. coli* 6.2E had 22%, 0%, 49%, 65% and 120% survival in 25% NHS, GO, GO-PcZr(Lys)_2_-Ag, GO-Ag, and GO-PcZr(Lys)_2_, respectively (as illustrated in Figure 1). This strain also showed 79% (*p* > 0.05), 19% (*p* = 0.0050), 32% (*p* = 0.0012), and 13% (*p* = 0.0091) survival in simultaneously mixed NHS with GO, GO-PcZr(Lys)_2_-Ag, GO-Ag, and GO-PcZr(Lys)_2_ nanocomposites, respectively. These results showed that one hour is not enough for nanocomposites GO-PcZr(Lys)_2_-Ag and GO-PcZr(Lys)_2_ simultaneously added to NHS and *E. coli* 6.2.E to block or stimulate the complement activity (as illustrated in Figure 1C). Only GO-PcZr(Lys)_2_-Ag nanocomposite had an enhanced bactericidal effect after IR irradiation (as illustrated in Figure 1D, *p* = 0.0058). Bacteria incubated in iNHS and the nanocomposite GO-Ag had similar survival (31% in iNHS and GO-Ag, data not shown) compared to active serum (32% in NHS and GO-Ag), which strongly suggests the significant role of the nanocomposite GO-Ag in the bactericidal effect (as illustrated in Figure 1C).

Further experiments included treatment for 30 min with a single component before mixing with the rest of them for 60 min, and their aim was to verify whether the treatment of a single component had an impact on the bacterial sensitivity to nanocomposites or human serum, respectively. NHS exposure to GO-PcZr(Lys)_2_-Ag and GO, and GO-Ag without IR irradiation had no statistically significant impact on bactericidal action. However, the NHS treatment of GO-PcZr(Lys)_2_ nanocomposite resulted in the decrease of bacterial serum survival (27% survival, *p* = 0.0416, as illustrated in Figure 2A). It showed that the nanocomponent GO-PcZr(Lys)_2_ had a more crucial role in the bactericidal effect and GO-PcZr(Lys)_2_-Ag might have inactivated the serum complement.

The insignificant reduced bactericidal ability shown by GO-PcZr(Lys)_2_-Ag-treated NHS (as illustrated in Figure 2A) was intensified by IR exposure (as illustrated in Figure 2C). In GO-PcZr(Lys)_2_-Ag-treated NHS that was subsequently irradiated, 43% bacterial survival was observed (*p* = 0.0142). In contrast, without IR irradiation survival was 98% (*p* > 0.05, no statistically significant difference between T0 and T60, as illustrated in Figure 2A). The role of light in reducing the number of bacteria was not observed in GO, GO-Ag, and GO-PcZr(Lys)_2_-exposed NHS (240%, 49% and 17% survival, respectively, after IR irradiation, as illustrated in Figure 2C, *p* > 0.05), because, without IR exposure the strain displayed a lower or similar survival rate (150%, 23%, and 27% after 60 min of incubation, as illustrated in Figure 2A). Summarizing the above, IR irradiation enhances bactericidal activity of serum in the case of the GO-PcZr(Lys)_2_.

This research showed that bacteria exposed to nanocomposites become more sensitive to serum action (*p* < 0.05, as illustrated in Figure 4A). Only 12%, 1%, and 1% of bacteria exposed to GO-PcZr(Lys)_2_-Ag, GO-Ag, and GO-PcZr(Lys)_2_ nanocomposites, respectively, survived the human serum activity. *E. coli* 6.2E showed high susceptibility to GO action with a survival rate of 0% after 30-min incubation before the serum treatment (as illustrated in Figure 4A). This observation could be explained by the fact of changes appearing in the bacterial outer membrane, which facilitate complement activity. However, these changes caused by the nanocomponents are not crucial enough for cells to multiply again in iNHS (as illustrated in Figure 4B).

Bacteria exposed to serum survived action of pure GO, GO-PcZr(Lys)_2_-Ag, and GO-PcZr(Lys)_2_ samples (*p* > 0.05, as illustrated in Figure 6A). The strain exhibited 68%, 46%, and 93% survival for GO-PcZr(Lys)_2_-Ag, GO-PcZr(Lys)_2_, and GO, respectively, after 60 min of the experiment. Compared to the strain not treated with serum there was 19%, 13%, and 79% survival in GO-PcZr(Lys)_2_-Ag, GO-PcZr(Lys)_2_, and the GO nanocomponent mixed with NHS, respectively (as illustrated in Figure 1C). In contrast, bacteria also exposed to serum become more sensitive to the GO-Ag sample (*p* < 0.0001, as illustrated in Figure 6A) based on survival rate changes from 32% in nonserum-treated conditions (as illustrated in Figure 1C) to 1% survival after serum treatment (as illustrated in Figure 6A).

In vivo cytotoxicity tests conducted for all designed graphite oxide nanocomposites in MIC concentrations revealed no toxic effect toward *G. mellonella* larvae. These results are the first step towards assessment of the suitability of these compounds for future testing with mammals and clinical use in humans. Cell cultures are used as an in vitro model for toxicity testing, but there is still a huge difference in comparison to that of whole animals [40]. *G. mellonella* larvae have the potential to predict the cytotoxic effects of various compounds in mammals [40,41,42]. The present study is the first one devoted to the examination of the cytotoxic activity of graphite oxide nanocomposites using *G. mellonella* larvae.

## 4. Materials and Methods

### 4.1. Bacterial Strains

In this study, *E. coli* 6.2E isolated from alveoli was used. This strain was provided by the Medical University of Lublin. The *E. coli* J53 (pMG101) silver-resistant *E. coli* K-12-J53 strain carrying the plasmid pMG101 from the National Collection of Type Cultures was used as a control in all experiments [43]. Results for this strain are presented in Appendix A.

### 4.2. Nanocomposites

**Figure 9:**
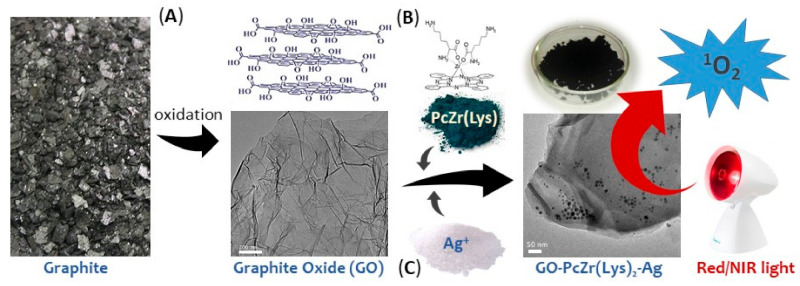
Scheme of GO-PcZr(Lys)_2_-Ag composite preparation. During first step of synthesis (**A**), reaction of graphite in strongly oxidizing conditions gives graphite oxide (GO). In next step (**B**), bis(lysinato)–PcZr is added in dimethylformamide and reaction proceeds in presence of dicyclohexylcarbodiimide used as a linker (GO-PcZr(Lys)_2_). Subsequently (**C**), silver nitrate is added to aqueous suspension of GO-PcZr(Lys)_2_ and Ag^+^ ions are reduced by the addition of ascorbic acid, resulting in a three-component composite (GO-(PcZr(Lys)_2_-Ag).

The following samples described in detail previously [9,29,44,45] were tested in this study: pure graphite oxide (GO), GO-PcZr(Lys)_2_-Ag (0.5 g/10 mL) (IV), GO-Ag (0.5 g/10 mL) (V), and GO-PcZr(Lys)_2_ (VI). A brief scheme of composites preparation is shown in Figure 9.

### 4.3. IR Exposure

Infrared irradiation was repeated five times, with 2 min of exposure, at a distance of 50 cm from opened Eppendorf tubes containing a mixture of NHS and nanocomposites, and a 1 min short break with closed tubes.

### 4.4. Normal Human Serum (NHS)

NHS (Sigma–Aldrich), sterile-filtered, contained macromolecules, carrier proteins, attachment and spreading factors, low molecular weight nutrients, and hormones and growth factors [46]. The serum was frozen in 0.5-mL and 1-mL aliquots at −70 °C for a period no longer than 2 months. Each aliquot of serum was used only once and thawed immediately before the experiment. Utilization of the residual NHS, iNHS and their mixtures with bacteria was carried out by the appropriate company, cooperating with the Department of Microbiology, Faculty of Biological Sciences, University of Wroclaw.

### 4.5. Inactivated Normal Human Serum (iNHS)

Serum inactivation was achieved by incubation at 56 °C for 30 min. The aim of this treatment was to confirm that the complement is responsible for the bactericidal action of NHS and confirmation of the multiplication of bacterial strains.

### 4.6. Serum Bactericidal Assay

The bactericidal activity of NHS was determined as described previously [47]. Briefly, LB broth (Biocorp) was inoculated to attain an optical density at 600 nm (OD_600_) of 0.1 with an overnight culture of *n* = 2 *E. coli* strains and incubated at 37 °C with shaking at 250 rpm in an incubator to OD_600_ 0.3. Then cells were collected by centrifugation at 4000 rpm for 20 min at 4 °C. The pellets were resuspended in 3 mL of saline (0.9% NaCl) and then diluted to a cell density of 10^6^ CFU/mL (colony forming units in 1 mL). Aliquots of the cell suspension were mixed with an equal volume of NHS or iNHS at a final concentration of 25% (*v*/*v*) and incubated at 37 °C for 0, 15, 30, and 60 min in an incubator with shaking at 250 rpm. The nanocomposites were added in MIC concentration: GO-PcZr(Lys)_2_-Ag with or without IR light 64 µg/mL; GO-Ag without IR light 512 µg/mL, and with IR light 128 µg/mL; GO-PcZr(Lys)_2_ and rGO with or without IR light 4096 µg/mL. Every treatment was conducted at 37 °C for 30 min prior the main serum bactericidal assay. The serial dilutions were plated onto LB agar (Biocorp) in duplicate or triplicate, incubated at 37 °C for 24 h, and CFU/mL of bacteria exposed to the serum was calculated.

### 4.7. Statistical Analysis

Bacterial survival data at the defined time points were averaged, transformed from CFU/mL values to percentage survival. The mean survival decrease or increase over time were studied using mixed ANOVA followed by Dunnett’s multiple comparisons test (within groups, comparing T0 and T60 survival values) or Tukey’s multiple comparisons test (between groups, comparing T60 survival values as presented in Appendix B; GraphPad Prism v. 9.1.1). Additionally, the resulting time series from all experiments were compared by calculating distances with the DTW algorithm and clustered using the fuzzy algorithm, both from the dtwclust 5.5.6 package in R [48]. Results for this analysis are presented in Appendix E. Missing values were interpolated using the imputeTS 3.1 package in R [49]. The fuzzy clusters were visualized using the linear projection tool from Orange 3 [50].

### 4.8. Galleria mellonella Treatment Assays

#### 4.8.1. *G. mellonella* Larvae Acquisition

*G. mellonella* larvae were obtained from the culture of larvae at the Department of Microbiology of University of Wrocław. Healthy larvae were selected as those possessing a cream color with minimal speckling and no grey markings, proper firmness and elasticity, high motility, about 250 mg in weight, and 2–3 cm in length [18,19]. Healthy larvae (*n* = 10) were selected and placed in separate Petri dishes.

#### 4.8.2. In Vivo Cytotoxicity Tests

To test the toxic effect of tested graphite oxide nanocomposites, larvae (*n* = 10) were injected with appropriate probes: GO-PcZr(Lys)_2_-Ag, GO-Ag, GO-PcZr(Lys)_2_, and GO, respectively. Compounds (10 μL) were administered into the hemocoels through the last left proleg using a 25 μL Hamilton syringe (Hamilton, Shanghai, People’s Republic of China). Larvae were incubated at 37 °C in the dark. Previously determined MIC concentrations of each tested nanocomposite were used for injection: GO-PcZr(Lys)_2_-Ag 64 µg/mL; GO-Ag 512 µg/mL; GO-PcZr(Lys)_2_ and rGO 4096 µg/mL. Ten inoculated (sterile PBS) larvae were used as controls. The larvae were observed for survival every 24 h for 5 days. Larvae were considered dead when no response was observed following touch [18,19,23]. Obtained data were pooled from a minimum of three independent experiments.

## 5. Conclusions

The following conclusions can be made for the *E. coli* 6.2E bacterial strain tested in this work:

- GO-PcZr(Lys)_2_, without IR irradiation enhance the antimicrobial efficacy of the human serum;
- IR irradiation enhances bactericidal activity of human serum in the case of the GO-PcZr(Lys)_2_-Ag sample;
- bacteria exposed to nanocomposites become more sensitive to the action of human serum;
- bacteria exposed to human serum become more sensitive to the GO-Ag sample;
- the influence of GO nanocomposites on the antibacterial activity of human serum and the impact on the bacterial sensitivity to human serum after their contact with GO nanocomposites strongly depend on the physicochemical properties of GO nanocomposites;
- the designed graphite nanocomposites showed no cytotoxic effect toward *Galleria mellonella* larvae;
- in comparison to that of *E. coli* J53, antimicrobial efficacy of human serum depends on individual properties of bacteria.

## Figures and Tables

**Table 1:** Survival of *G. mellonella* larvae during 24 h after application of graphite nanocomposites in MIC concentration: GO-PcZr(Lys)_2_-Ag 64 µg/mL; GO-Ag 512 µg/mL; GO- PcZr(Lys)_2_ and rGO 4096 µg/mL.

	Larvae Survival in 24 h after Nanocomposite Administration Number of Live Larvae [*n*] (Percentage of Live Larvae [%])			
Nanocomposites	Run 1	Run 2	Run 3	Medium
GO-PcZr(Lys)_2_-Ag (IV)	9 (90%)	10 (100%)	9 (90%)	9.3 (93%)
GO-Ag (V)	10 (100%)	10 (100%)	10 (100%)	10 (100%)
GO-PcZr(Lys)_2_ (VI)	9 (90%)	10 (100%)	10 (100%)	9.6 (96%)
rGO	8 (100%)	10 (100%)	9 (100%)	9 (90%)

**Figure A1:**
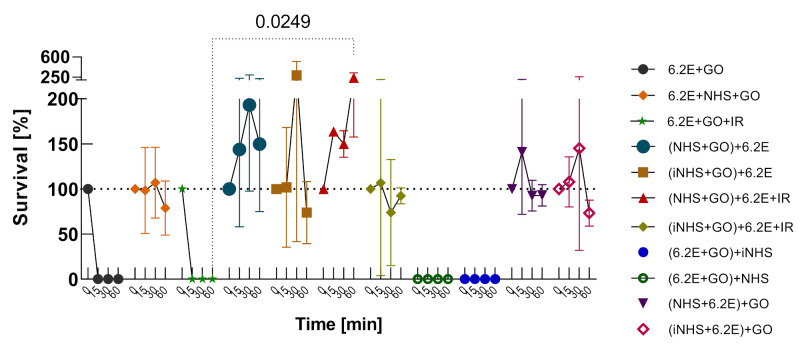
All types of bacterial survival experiments involving GO and *E. coli* 6.2E presented altogether. Graphs represent percentage of live bacteria in sample relative to average number at time zero. Standard deviation values are presented with error bars, Tukey’s test *p*-values for between group T60 pairs are shown above graph when equal or lower than 0.05. Corresponding results obtained for strain *E. coli J53* are presented in Figure A9.

**Figure A2:**
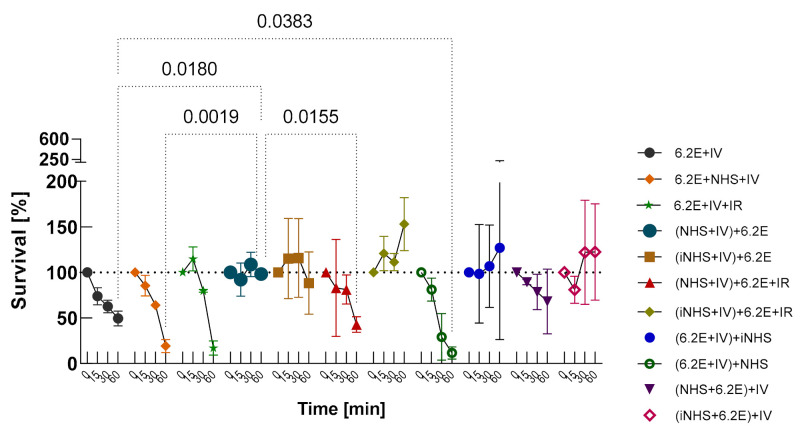
All types of bacterial survival experiments involving nanocomposite IV: GO-PcZr(Lys)_2_-Ag and *E. coli* 6.2E. presented altogether. Graphs represent percentage of live bacteria in sample relative to the average number at time zero. Standard deviation values are presented with error bars, Tukey’s test *p*-values for between group T60 pairs are shown above graph when equal or lower than 0.05. Corresponding results obtained for strain *E. coli J53* are presented in Figure A10.

**Figure A3:**
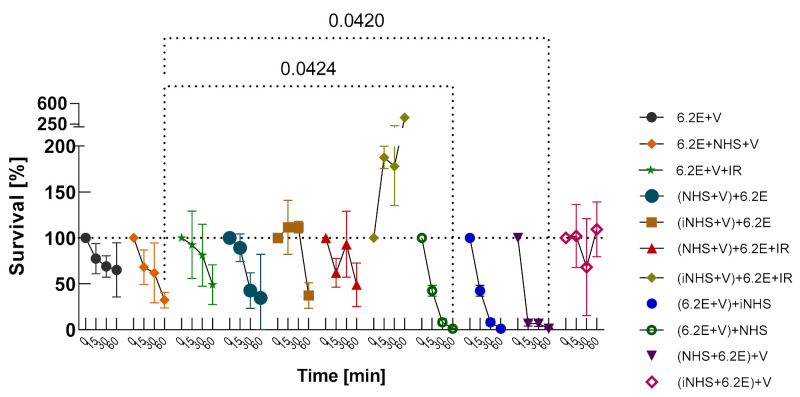
All types of bacterial survival experiments involving nanocomposite V: GO-Ag and *E. coli* 6.2E presented altogether. Graphs represent percentage of live bacteria in sample relative to average number at time zero. Standard deviation values are presented with error bars, Tukey’s test *p*-values for between group T60 pairs are shown above graph when equal or lower than 0.05. Corresponding results obtained for strain *E. coli J53* are presented in Figure A11.

**Figure A4:**
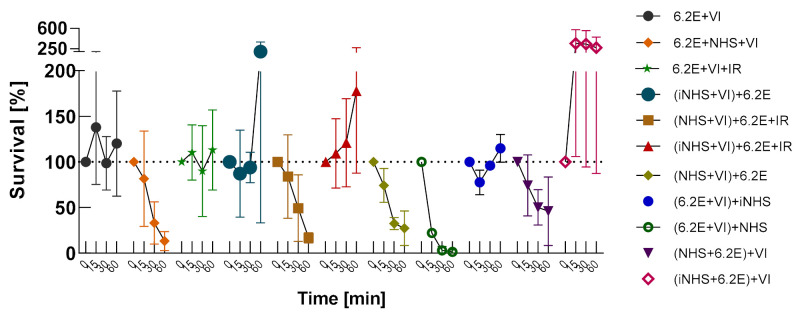
All types of bacterial survival experiments involving nanocomposite VI: GO-PcZr(Lys)_2_ and *E. coli* 6.2E presented altogether. Graphs represent percentage of live bacteria in sample relative to the average number at time zero. Standard deviation values are presented with error bars, Tukey’s test *p*-values for between group T60 pairs are shown above graph when equal or lower than 0.05. Corresponding results obtained for strain *E. coli J53* are presented in Figure A12.

**Figure A5:**
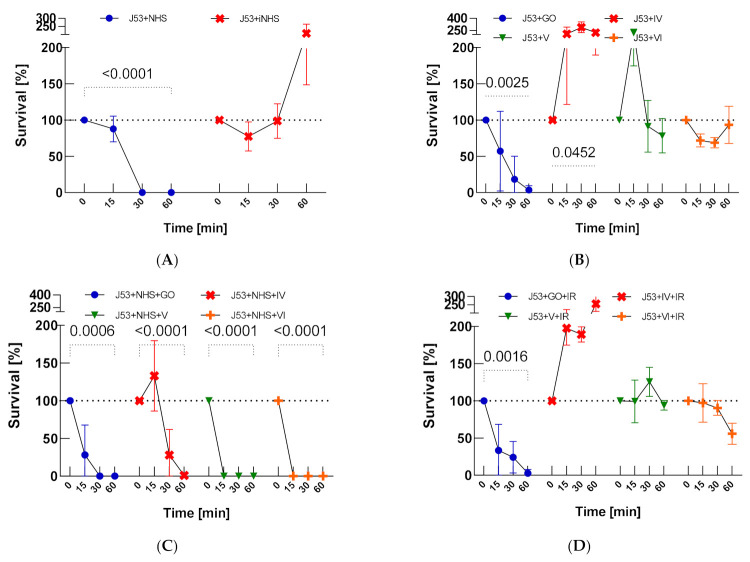
Bacterial survival in NHS and iNHS (**A**), nanocomposites GO, IV: GO-PcZr(Lys)_2_-Ag, V: GO-Ag, VI: GO-PcZr(Lys)_2_ without IR irradiation (**B**) nanocomposites GO, IV: GO-PcZr(Lys)_2_-Ag, V: GO-Ag, VI: GO-PcZr(Lys)_2_ with IR irradiation (**D**) and mixed NHS with nanocomposites (**C**). Graphs represent percentage of live bacteria in sample relative to average number at time zero. Standard deviation values are presented with error bars, Dunnett’s test *p*-values for within group T0-T60 pairs are shown above graph when equal or lower than 0.05.

**Figure A6:**
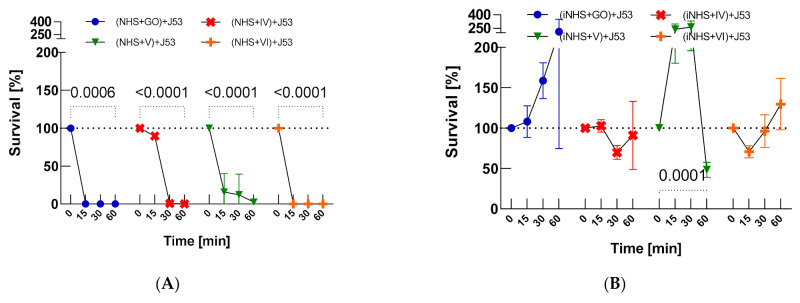

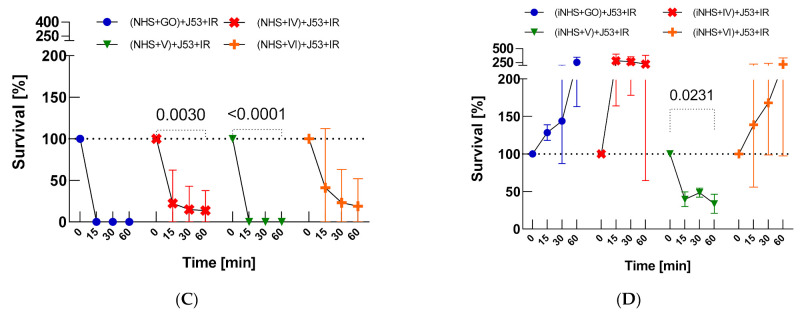
Bactericidal effectiveness of normal human serum exposed to nanocomposites (GO, IV: GO-PcZr(Lys)_2_-Ag, V: GO-Ag, VI: GO-PcZr(Lys)_2_) before (**A**) and after IR irradiation (**C**), the control test in iNHS (**B**,**D**). Graphs represent percentage of live bacteria in sample relative to average number at time zero. Standard deviation values are presented with error bars, Dunnett’s test *p*-values for within group T0–T60 pairs are shown above graph when equal or lower than 0.05.

**Figure A7:**
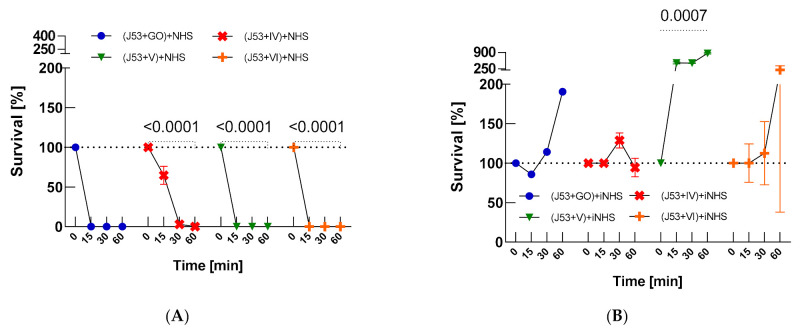
Serum sensitivity of bacteria exposed to nanocomposites (GO, IV: GO-PcZr(Lys)_2_-Ag, V: GO-Ag, VI: GO-PcZr(Lys)_2_) (**A**) and control test in iNHS (**B**). Graphs represent percentage of live bacteria in the sample relative to average number at time zero. Standard deviation values are presented with error bars, Dunnett’s test *p*-values for within group T0–T60 pairs are shown above graph when equal or lower than 0.05.

**Figure A8:**
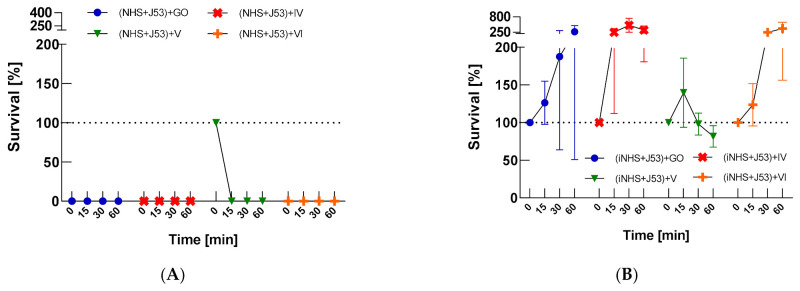
Sensitivity of bacteria to nanocomposites (GO, IV: GO-PcZr(Lys)_2_-Ag, V: GO-Ag, VI: GO-PcZr(Lys)_2_) after exposure to serum (**A**) and control test in iNHS (**B**). Graphs represent percentage of live bacteria in sample relative to average number at time zero. Standard deviation values are presented with error bars, Dunnett’s test *p*-values for within group T0–T60 pairs are shown above graph when equal or lower than 0.05.

**Figure A9:**
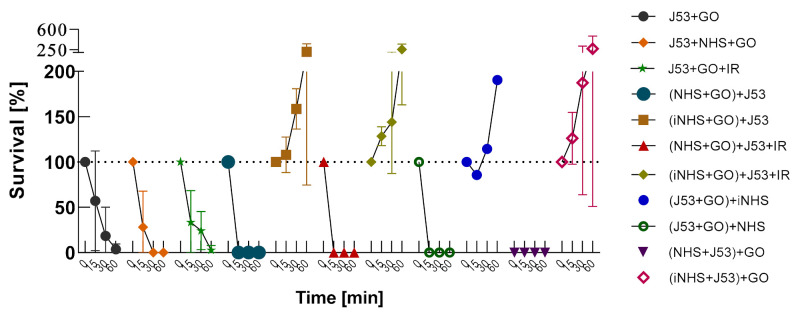
All types of bacterial survival experiments involving GO and *E. coli* J53. presented altogether. Graphs represent percentage of live bacteria in sample relative to average number at time zero. Standard deviation values are presented with error bars, Tukey’s test *p*-values for between group T60 pairs are presented in Table A2.

**Figure A10:**
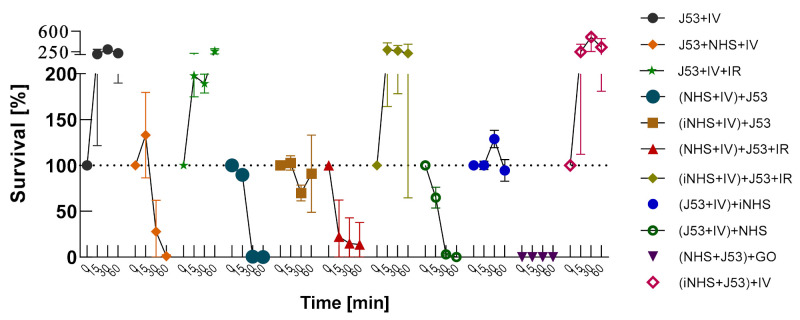
All types of bacterial survival experiments involving nanocomposite IV: GO-PcZr(Lys)_2_-Ag and *E. coli* J53. presented altogether. Graphs represent percentage of live bacteria in sample relative to average number at time zero. Standard deviation values are presented with error bars, Tukey’s test *p*-values for between group T60 pairs are presented in Table A2.

**Figure A11:**
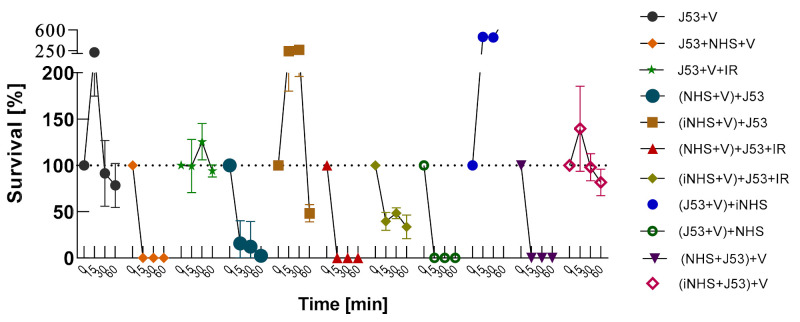
All types of bacterial survival experiments involving nanocomposite V: GO-Ag and *E. coli* J53. presented altogether. Graphs represent percentage of live bacteria in sample relative to average number at time zero. Standard deviation values are presented with error bars, Tukey’s test *p*-values for between group T60 pairs are presented in Table A2.

**Figure A12:**
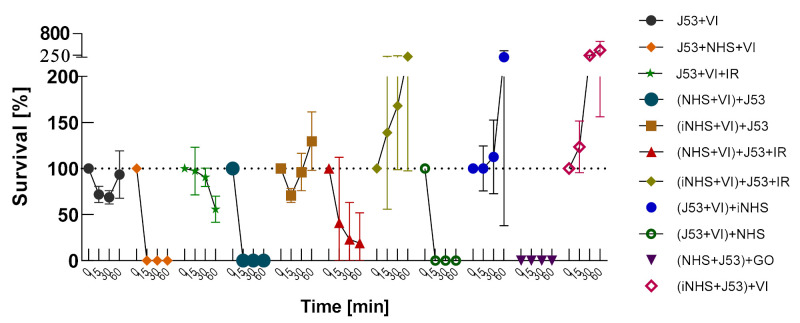
All types of bacterial survival experiments involving nanocomposite VI: GO-PcZr(Lys)_2_ and *E. coli* J53. presented altogether. Graphs represent percentage of live bacteria in the sample relative to average number at time zero. Standard deviation values are presented with error bars, Tukey’s test *p*-values for between group T60 pairs are presented in Table A2.

**Figure A13:**
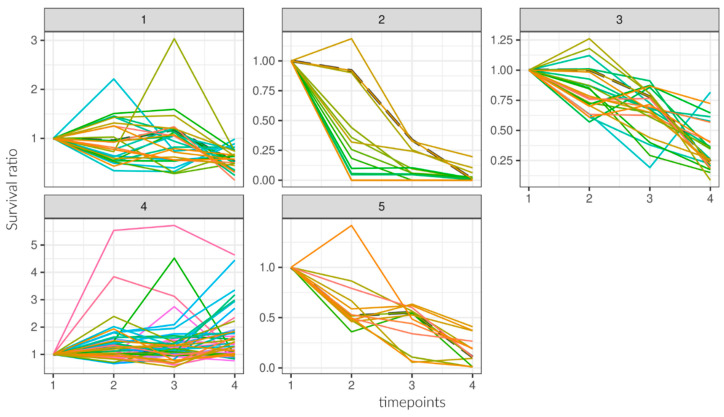
*Escherichia coli* 6.2E survival experiments results represented as graphs and divided into 5 clusters using DTW/fuzzy clustering algorithms. Cluster 1 was named as ‘Weak growth/decline’, cluster 2 as ‘Rapid decline’, cluster 3 as ‘Slight decline’, cluster 4 as ‘Growth’ and cluster 5 as ‘Moderate decline’. Each line represents one experiment replicate. Dashed line is the centroid of cluster.

## Data Availability

Not applicable.

## Appendix A

**Table A1:** Bacterial survival experiments results expressed as CFU/mL values, measured in four timepoints (0, 15, 30, and 60 min).

Replicate	T0	T15	T30	T60
6.2E+NHS	582,000,000	728,000,000	61,000,000	178,000,000
6.2E+NHS_2	795,000,000	603,000,000	608,000,000	153,000,000
6.2E+NHS_3	58,000,000	535,000,000	718,000,000	8,500,000
6.2E+iNHS	463,000,000	623,000,000	453,000,000	545,000,000
6.2E+iNHS_2	573,000,000	493,000,000	383,000,000	825,000,000
6.2E+iNHS_3	508,000,000	493,000,000	355,000,000	84,000,000
6.2E+GO	62,000	0	0	0
6.2E+GO_2	51,250	0	0	0
6.2E+GO_3	76,000	0	0	0
6.2E+IV	3,680,000	2,330,000	2,300,000	1,500,000
6.2E+IV_2	4,580,000	3,530,000	3,180,000	2,600,000
6.2E+IV_3	4,680,000	3,800,000	2,600,000	2,370,000
6.2E+V	3,330,000	2,030,000	2,380,000	1,330,000
6.2E+V_2	2,850,000	2,200,000	1,600,000	1,650,000
6.2E+V_3	2,000,000	1,880,000	1,580,000	1,950,000
6.2E+VI	4,330,000	8,380,000	3,330,000	4,500,000
6.2E+VI_2	3,670,000	5,500,000	4,830,000	6,750,000
6.2E+VI_3	7,380,000	5,170,000	6,400,000	5,330,000
6.2E+NHS+GO	3,830,000	4,830,000	5,170,000	4,330,000
6.2E+NHS+GO_2	9,630,000	4,170,000	7,630,000	6,330,000
6.2E+NHS+GO_3	8,130,000	10,200,000		4,670,000
6.2E+NHS+IV	655,000,000	645,000,000	438,000,000	145,000,000
6.2E+NHS+IV_2	713,000,000	560,000,000	463,000,000	173,000,000
6.2E+NHS+IV_3	680,000,000	538,000,000	408,000,000	75,000,000
6.2E+NHS+V	603,000,000	298,000,000	205,000,000	160,000,000
6.2E+NHS+V_2	735,000,000	520,000,000	323,000,000	193,000,000
6.2E+NHS+V_3	580,000,000	540,000,000	623,000,000	185,000,000
6.2E+NHS+V_4	718,000,000	423,000,000	445,000,000	320,000,000
6.2E+NHS+VI	6,630,000	3,500,000	2,920,000	1,290,000
6.2E+NHS+VI_2	6,000,000	8,500,000	2,920,000	1,140,000
6.2E+NHS+VI_3	8,000,000	3,990,000	525,000	100,000
6.2E+GO+IR	2,333,333	0	0	0
6.2E+GO+IR_2	25,700	0	0	0
6.2E+GO+IR_3	19,500	0	0	0
6.2E+IV+IR	2,670,000	2,680,000	2,080,000	650,000
6.2E+IV+IR_2	2,300,000	2,900,000	1,850,000	200,000
6.2E+IV+IR_3	2,230,000	2,630,000	1,800,000	400,000
6.2E+V+IR	2,000,000	2,630,000	2,400,000	1,480,000
6.2E+V+IR_2	2,470,000	1,450,000	1,530,000	920,000
6.2E+V+IR_3	2,500,000	2,180,000	1,530,000	900,000
6.2E+VI+IR	5,250,000	7,500,000	7,700,000	3,330,000
6.2E+VI+IR_2	6,750,000	7,080,000	4,670,000	8,700,000
6.2E+VI+IR_3	5,880,000	4,880,000	3,170,000	8,630,000
(NHS+GO)+6.2E	51,250	61,250	67,500	78,750
(NHS+GO)+6.2E_2	45,000	107,500	65,000	100,000
(NHS+GO)+6.2E_3	118,000	86,000	358,000	86,250
(NHS+IV)+6.2E	6,100,000	4,330,000	7,450,000	5,630,000
(NHS+IV)+6.2E_2	7,800,000	8,030,000	8,430,000	7,880,000
(NHS+IV)+6.2E_3	5,950,000	6,080,000	5,680,000	6,000,000
(NHS+V)+6.2E	5,280,000	4,850,000	1,780,000	50,000
(NHS+V)+6.2E_2	6,720,000	4,900,000	4,350,000	0
(NHS+V)+6.2E_3	5,870,000	6,030,000	1,730,000	4,000,000
(NHS+VI)+6.2E	88,000	75,000	25,750	13,300
(NHS+VI)+6.2E_2	6,333,333	3,333,333	17,800	31,000
(NHS+VI)+6.2E_3	72,500	61,250	29,000	12,625
(iNHS+GO)+6.2E	9,100,000	5,000,000	10,200,000	4,500,000
(iNHS+GO)+6.2E_2	9,800,000	14,600,000	44,300,000	9,630,000
(iNHS+IV)+6.2E	475,000,000	665,000,000	510,000,000	410,000,000
(iNHS+IV)+6.2E_2	662,000,000	475,000,000	578,000,000	427,000,000
(iNHS+IV)+6.2E_3	388,000,000	585,000,000	618,000,000	297,000,000
(iNHS+IV)+6.2E_4	253,000,000	410,000,000	425,000,000	393,000,000
(iNHS+IV)+6.2E_5	395,000,000	443,000,000	470,000,000	330,000,000
(iNHS+IV)+6.2E_6	583,000,000	318,000,000	315,000,000	370,000,000
(iNHS+V)+6.2E	535,000,000	510,000,000	630,000,000	278,000,000
(iNHS+V)+6.2E_2	398,000,000	373,000,000	418,000,000	140,000,000
(iNHS+V)+6.2E_3	347,000,000	505,000,000	390,000,000	85,000,000
(iNHS+VI)+6.2E	9,380,000	5,000,000	7,700,000	7,700,000
(iNHS+VI)+6.2E_2	8,900,000	10,800,000	9,400,000	28,300,000
(NHS+GO)+6.2E+IR	95,000		132,500	284,000
(NHS+GO)+6.2E+IR_2	3,666,667	60,000	58,750	6,666,667
(NHS+IV)+6.2E+IR	4,350,000	2,480,000	3,730,000	1,530,000
(NHS+IV)+6.2E+IR_2	4,670,000	6,730,000	4,400,000	2,430,000
(NHS+IV)+6.2E+IR_3	4,100,000	1,950,000	2,600,000	1,680,000
(NHS+V)+6.2E+IR	3,980,000	1,830,000	2,100,000	1,470,000
(NHS+V)+6.2E+IR_2	3,470,000	2,680,000	4,200,000	1,170,000
(NHS+V)+6.2E+IR_3	2,530,000	1,580,000	2,670,000	1,930,000
(NHS+VI)+6.2E+IR	7,166,667	85,000	2,333,333	14,100
(NHS+VI)+6.2E+IR_2	101,000	102,000	92,000	20,500
(NHS+VI)+6.2E+IR_3	75,000	24,000	18,250	7,833,333
(iNHS+GO)+6.2E+IR	24,200,000	8,250,000	7,800,000	23,800,000
(iNHS+GO)+6.2E+IR_2	8,130,000	14,600,000	9,380,000	7,000,000
(iNHS+IV)+6.2E+IR	370,000,000	368,000,000	373,000,000	687,000,000
(iNHS+IV)+6.2E+IR_2	370,000,000	500,000,000	423,000,000	530,000,000
(iNHS+IV)+6.2E+IR_3	398,000,000	508,000,000	473,000,000	517,000,000
(iNHS+V)+6.2E+IR	180,000,000	363,000,000	233,000,000	530,000,000
(iNHS+V)+6.2E+IR_2	178,000,000	323,000,000	347,000,000	597,000,000
(iNHS+V)+6.2E+IR_3	153,000,000	275,000,000	320,000,000	680,000,000
(iNHS+VI)+6.2E+IR	6,170,000	8,630,000	10,800,000	10,900,000
(iNHS+VI)+6.2E+IR_2	8500,000	5,670,000	7,000,000	22,800,000
(iNHS+VI)+6.2E+IR_3	7500,000	9,100,000	7,900,000	6,630,000
(6.2E+GO)+NHS	0	0	0	0
(6.2E+GO)+NHS_2	0	0	0	0
(6.2E+IV)+NHS	270,000,000	180,000,000	15,000,000	25,500,000
(6.2E+IV)+NHS_2	353,000,000	318,000,000	90,000,000	21,800,000
(6.2E+IV)+NHS_3	177,000,000	153,000,000	100,000,000	33,500,000
(6.2E+V)+NHS	265,000,000	117,000,000	15,000,000	4,000,000
(6.2E+V)+NHS_2	232,000,000	110,000,000	25,000,000	2,000,000
(6.2E+V)+NHS_3	298,000,000	107,000,000	0	3,000,000
(6.2E+VI)+NHS	7,130,000	1,840,000	425,000	200,000
(6.2E+VI)+NHS_2	7,000,000	1,290,000	0	0
(6.2E+GO)+iNHS	0	0	0	0
(6.2E+GO)+iNHS_2	0	0	0	0
(6.2E+GO)+iNHS_3	0	0	0	0
(6.2E+IV)+iNHS	325,000,000	168,000,000	18,000,000	3,500,000
(6.2E+IV)+iNHS_2	348,000,000	298,000,000	488,000,000	623,000,000
(6.2E+IV)+iNHS_3	144,000,000	227,000,000	18,000,000	275,000,000
(6.2E+V)+iNHS	265,000,000	117,000,000	1,500,000	40,000
(6.2E+V)+iNHS_2	232,000,000	11,000,000	2,500,000	20,000
(6.2E+V)+iNHS_3	298,000,000	107,000,000	0	30,000
(6.2E+VI)+iNHS	63,750,000	43,333,330,000	6,250,000	80,000
(6.2E+VI)+iNHS_2	61,250,000	53,333,330,000	5,750,000	63,750,000
(NHS+6.2E)+GO	180,000	18,333,330,000	1,950,000	190,000
(NHS+6.2E)+GO_2	19,250,000	42,583,330,000	14,333,330,000	1,580,000
(NHS+6.2E)+GO_3	1,830,000	183,750,000	1740,000	1,670,000
(NHS+6.2E)+IV	365,000,000	338,000,000	245,000,000	133,000,000
(NHS+6.2E)+IV_2	375,000,000	327,000,000	253,000,000	23,000,000
(NHS+6.2E)+IV_3	233,000,000	20,500,0000	235,000,000	248,000,000
(NHS+6.2E)+V	43,000,000	42,500,000	4,500,000	650,000
(NHS+6.2E)+V_2	58,000,000	32,500,000	300,000	90,000
(NHS+6.2E)+V_3	533,000,000	2,500,000	2,500,000	250,000
(NHS+6.2E)+VI	1,240,000	1,390,000	9000	325,000
(NHS+6.2E)+VI_2	3,520,000	1,740,000	140,000	3,140,000
(NHS+6.2E)+VI_3	860,000	5,250,000	32,666,670,000	1,910,000
(iNHS+6.2E)+GO	840,000	730,000	5,750,000	48,333,330,000
(iNHS+6.2E)+GO_2	91,250,000	12,700,000	83,750,000	70,000
(iNHS+6.2E)+GO_3	950,000	9,250,000	260,833,300,000	81,250,000
(iNHS+6.2E)+IV	743,000,000	478,000,000	417,000,000	477,000,000
(iNHS+6.2E)+IV_2	413,000,000	383,000,000	653,000,000	69,000,000
(iNHS+6.2E)+IV_3	402,000,000	345,000,000	61,000,000	547,000,000
(iNHS+6.2E)+V	467,000,000	605,000,000	58,000,000	658,000,000
(iNHS+6.2E)+V_2	692,000,000	44,000,000	133,000,000	565,000,000
(iNHS+6.2E)+V_3	402,000,000	455,000,000	245,000,000	423,000,000
(iNHS+6.2E)+VI	166,250,000	920,000	950,000	770,000
(iNHS+6.2E)+VI_2	68,333,330,000	262,500,000	213,571,400,000	7,250,000
(iNHS+6.2E)+VI_3	960,000	84,166,670,000	990,000	225,714,300,000
J53+NHS	7,170,000	6,400,000	0	0
J53+NHS_2	7,950,000	5,500,000	0	0
J53+NHS_3	7,120,000	7,450,000	0	0
J53+iNHS	6,230,000	6,130,000	7,830,000	170,000,000
J53+iNHS_2	7,900,000	5,950,000	6,380,000	1,450,000,000
J53+iNHS_3	7,320,000	4,280,000	6,580,000	120,000,000
J53+GO	143,000	1,566,667	78,750	15,000
J53+GO_2	140,000	0	0	0
J53+GO_3	102,500	6,333,333	0	0
J53+IV	5,050,000	1,540,000,000	1,830,000,000	1,290,000,000
J53+IV_2	6,830,000	1,140,000,000	1,860,000,000	1,290,000,000
J53+IV_3	7,250,000	1,080,000,000	1,680,000,000	1,630,000,000
J53+V	8,750,000	2,390,000,000	4,430,000	4,750,000
J53+V_2	5,750,000	1,050,000,000	6,130,000	5,850,000
J53+V_3	7,580,000	1,580,000,000	8,850,000	6,000,000
J53+VI	1,583,333	130,000	106,250	193,000
J53+VI_2	6,666,667	45,000	4,166,667	57,500
J53+VI_3	129,000	85,000	98,750	93,000
J53+NHS+GO	85,000	0	0	0
J53+NHS+GO_2	0	0	0	0
J53+NHS+GO_3	109,000	61,250	0	0
J53+NHS+IV	5,580,000	1,090,000,000	1,900,000	7500
J53+NHS+IV_2	7,720,000	1,280,000,000	6,230,000	155,000
J53+NHS+IV_3	8,350,000	1,310,000,000	4,400,000	305,000
J53+NHS+IV_4	4,730,000	3,830,000	0	0
J53+NHS+IV_5	6,200,000	7,030,000	0	0
J53+NHS+IV_6	7,620,000	6,530,000	0	0
J53+NHS+V	6,130,000	0	0	0
J53+NHS+V_2	7,770,000	0	0	0
J53+NHS+V_3	7,340,000	0	0	0
J53+NHS+V_4	7,330,000	0	0	0
J53+NHS+VI	138,000	0	0	0
J53+NHS+VI_2	8,166,667	0	0	0
J53+NHS+VI_3	75,000	0	0	0
J53+GO+IR	1,991,667	139,000	67,500	1,666,667
J53+GO+IR_2	1,383,333	0	0	0
J53+GO+IR_3	116,250	35,000	45,000	0
J53+IV+IR	7,400,000	1,330,000,000	1,350,000,000	2,010,000,000
J53+IV+IR_2	6,370,000	1,210,000,000	1,280,000,000	1,850,000,000
J53+IV+IR_3	7,870,000	1,760,000,000	1,450,000,000	1,610,000,000
J53+V+IR	6,870,000	6,300,000	7,750,000	6,000,000
J53+V+IR_2	4,950,000	6,480,000	7,330,000	4,960,000
J53+V+IR_3	6,300,000	4,730,000	7,300,000	5,940,000
J53+VI+IR	1,733,333	145,000	139,000	83,000
J53+VI+IR_2	74,000	60,000	67,500	35,000
J53+VI+IR_3	60,000	76,250	60,000	4,333,333
(NHS+GO)+J53	129,000	0	0	0
(NHS+GO)+J53_2	0	0	0	0
(NHS+GO)+J53_3	154,000	0	0	0
(NHS+IV)+J53	7,970,000	6,930,000	75,000	0
(NHS+IV)+J53_2	8,350,000	7,480,000	25,000	0
(NHS+IV)+J53_3	8,630,000	7,980,000	50,000	0
(NHS+V)+J53	6,200,000	0	0	0
(NHS+V)+J53_2	6,100,000	0	0	0
(NHS+V)+J53_3	7,080,000	0	0	0
(NHS+V)+J53_4	7,530,000	0	0	0
(NHS+V)+J53_5	7,430,000	3,480,000		550,000
(NHS+V)+J53_6	6,800,000	3,250,000	4,130,000	500,000
(NHS+VI)+J53	2,058,333	0	0	0
(NHS+VI)+J53_2	114,000	0	0	0
(NHS+VI)+J53_3	87,500	0	0	0
(iNHS+GO)+J53	237,500	2,466,667		2,333,333
(iNHS+GO)+J53_2	105,000	1,358,333	150,000	3,833,333
(iNHS+GO)+J53_3	83,750	760,00	146,000	145,000
(iNHS+IV)+J53	6,870,000	6,500,000	5,400,000	8,880,000
(iNHS+IV)+J53_2	7,300,000	7,550,000	4,500,000	3,350,000
(iNHS+IV)+J53_3	6,250,000	6,880,000	4,330,000	6,100,000
(iNHS+V)+J53	5,700,000	1,490,000,000	1,870,000,000	3,250,000
(iNHS+V)+J53_2	7,830,000	1,280,000,000	1,950,000,000	2,600,000
(iNHS+V)+J53_3	7,080,000	1,290,000,000	1,290,000,000	3,600,000
(iNHS+V)+J53_4	6,350,000	1,550,000,000	1,260,000,000	2,700,000
(iNHS+V)+J53_5	4,480,000	1,460,000,000	1,570,000,000	2,150,000
(iNHS+V)+J53_6	4,650,000	1,200,000,000	1,270,000,000	2,700,000
(iNHS+VI)+J53	1,666,667	132,000	123,750	155,000
(iNHS+VI)+J53_2	8,666,667	5,833,333	99,000	130,000
(iNHS+VI)+J53_3	89,000	5,833,333	89,000	130,000
(NHS+GO)+J53+IR	0	0	0	0
(NHS+GO)+J53+IR_2	0	0	0	0
(NHS+GO)+J53+IR_3	9,083,333	0	0	0
(NHS+IV)+J53+IR	5,420,000	5,000,000	3,500,000	3,010,000
(NHS+IV)+J53+IR_2	5,200,000	1,000,000	500,000	653,000
(NHS+IV)+J53+IR_3	6,000,000	0	0	0
(NHS+IV)+J53+IR_4	5,180,000	0	0	0
(NHS+IV)+J53+IR_5	6,100,000	0	0	0
(NHS+V)+J53+IR	333,000	0	0	0
(NHS+V)+J53+IR_2	133,000	0	0	0
(NHS+V)+J53+IR_3	533,000	0	0	0
(NHS+VI)+J53+IR	157,500	0	0	0
(NHS+VI)+J53+IR_2	90,000	1,108,333	62,500	51,250
(NHS+VI)+J53+IR_3	82,500	0	0	0
(iNHS+GO)+J53+IR	218,000	256,250	175,000	5,166,667
(iNHS+GO)+J53+IR_2	99,000	137,000	187,000	350,000
(iNHS+GO)+J53+IR_3	102,000	132,000	1,658,333	1,766,667
(iNHS+IV)+J53+IR	6,600,000	2,700,000,000	2,650,000,000	6,930,000
(iNHS+IV)+J53+IR_2	6,570,000	2,350,000,000	2,300,000,000	4,700,000
(iNHS+IV)+J53+IR_3	6,830,000	1,300,000,000	1,450,000,000	6,730,000
(iNHS+IV)+J53+IR_4	1,590,000,000	5,950,000,000	3,750,000,000	7,200,000,000
(iNHS+IV)+J53+IR_5	1,680,000,000	4,450,000,000	3,850,000,000	6,000,000,000
(iNHS+IV)+J53+IR_6	2,030,000,000	2,100,000,000	3,500,000,000	5,000,000,000
(iNHS+V)+J53+IR	4,980,000,000	1,430,000,000	2,100,000,000	2,400,000,000
(iNHS+V)+J53+IR_2	3,650,000,000	1,730,000,000	1,780,000,000	1,000,000,000
(iNHS+V)+J53+IR_3	3,980,000,000	1,700,000,000	2,150,000,000	1,000,000,000
(iNHS+VI)+J53+IR	173,000	8,916,667	1,683,333	164,000
(iNHS+VI)+J53+IR_2	4,666,667	101,250	110,000	155,000
(iNHS+VI)+J53+IR_3	71,000	105,000	1,216,667	157,500
(J53+GO)+NHS	10,000	0	0	0
(J53+GO)+NHS_2	0	0	0	0
(J53+GO)+NHS_3	0	0	0	0
(J53+IV)+NHS	7,500,000	4,200,000	147,000	0
(J53+IV)+NHS_2	7,780,000	4,730,000	280,000	0
(J53+IV)+NHS_3	7,470,000	5,800,000	237,000	0
(J53+V)+NHS	2,170,000	0	0	0
(J53+V)+NHS_2	2,300,000	0	0	0
(J53+V)+NHS_3	2,920,000	0	0	0
(J53+VI)+NHS	136,000	0	0	0
(J53+VI)+NHS_2	65,000	0	0	0
(J53+VI)+NHS_3	4,333,333	0	0	0
(J53+GO)+iNHS	0	0	10,000	20,000
(J53+GO)+iNHS_2	17,500	150,00	20,000	3,333,333
(J53+GO)+iNHS_3	0	0	0	0
(J53+IV)+iNHS	6,700,000	6,380,000	8,270,000	6,350,000
(J53+IV)+iNHS_2	6,420,000	6,680,000	8,970,000	5,300,000
(J53+IV)+iNHS_3	6,200,000	6,250,000	7,630,000	6,580,000
(J53+V)+iNHS	3,020,000	1,420,000,000	1,440,000,000	2,650,000,000
(J53+V)+iNHS_2	3,220,000	1,450,000,000	1,550,000,000	270,000,000
(J53+V)+iNHS_3	2,930,000	1,550,000,000	1,350,000,000	260,000,000
(J53+VI)+iNHS	1,708,333	1,466,667	1,566,667	147,500
(J53+VI)+iNHS_2	80,000	102,500	127,000	256,000
(J53+VI)+iNHS_3	81,250	70,000	71,000	
(NHS+J53)+GO	0	0	0	0
(NHS+J53)+GO_2	0	0	0	0
(NHS+J53)+GO_3	0	0	0	0
(NHS+J53)+IV	0	0	0	0
(NHS+J53)+IV_2	0	0	0	0
(NHS+J53)+IV_3	0	0	0	0
(NHS+J53)+V	2,680,000	0	0	0
(NHS+J53)+V_2	3,250,000	0	0	0
(NHS+J53)+V_3	5,080,000	0	0	0
(NHS+J53)+VI	0	0	0	0
(NHS+J53)+VI_2	0	0	0	0
(NHS+J53)+VI_3	0	0	0	0
(iNHS+J53)+GO	1,400,000	1,483,333	1,400,000	1,600,000
(iNHS+J53)+GO_2	194,000	284,000	5,333,333	8,166,667
(iNHS+J53)+IV	8,100,000	1,970,000,000	3,900,000,000	3,700,000,000
(iNHS+J53)+IV_2	3,630,000	1,380,000,000	2,750,000,000	1,300,000,000
(iNHS+J53)+IV_3	1,430,000,000	1,620,000,000	3,760,000,000	2,400,000,000
(iNHS+J53)+V	1,910,000,000	1,970,000,000	1,970,000,000	1,700,000,000
(iNHS+J53)+V_2	1,600,000,000	3,060,000,000	1,750,000,000	1,450,000,000
(iNHS+J53)+V_3	1,840,000,000	2,290,000,000	1,500,000,000	1,200,000,000
(iNHS+J53)+VI	1,458,333	1,791,667		186,250
(iNHS+J53)+VI_2	160,000	1,533,333	400,000	7,333,333
(iNHS+J53)+VI_3	98,750	150,000	235,000	550,000

## Appendix C

**Table A2:** Tukey’s test *p*-values > 0.05 for between group T60 pairs from bacterial survival experiments which results are represented in Figure A9, Figure A10, Figure A11 and Figure A12.

Experiments Comparison	*p*-Value
J53+IV vs. J53+NHS+IV	0.0369
J53+IV vs. (NHS+IV)+J53	0.0367
J53+IV vs. (NHS+IV)+J53+IR	0.0141
J53+IV vs. (J53+IV)+NHS	0.0367
J53+NHS+IV vs. (J53+IV)+iNHS	0.025
J53+IV+IR vs. (NHS+IV)+J53+IR	0.033
(NHS+IV)+J53 vs. (J53+IV)+iNHS	0.0255
(NHS+IV)+J53+IR vs. (J53+IV)+iNHS	0.011
(J53+IV)+NHS vs. (J53+IV)+iNHS	0.0255
J53+V vs. (J53+V)+iNHS	<0.0001
J53+NHS+V vs. J53+V+IR	0.0103
J53+NHS+V vs. (iNHS+V)+J53	0.0008
J53+NHS+V vs. (J53+V)+iNHS	<0.0001
J53+V+IR vs. (NHS+V)+J53	0.003
J53+V+IR vs. (iNHS+V)+J53	0.0028
J53+V+IR vs. (NHS+V)+J53+IR	0.0103
J53+V+IR vs. (iNHS+V)+J53+IR	0.0429
J53+V+IR vs. (J53+V)+NHS	0.0103
J53+V+IR vs. (J53+V)+iNHS	<0.0001
J53+V+IR vs. (NHS+J53)+V	0.0103
(NHS+V)+J53 vs. (iNHS+V)+J53	0.0003
(NHS+V)+J53 vs. (J53+V)+iNHS	<0.0001
(NHS+V)+J53 vs. (iNHS+J53)+V	0.0491
(iNHS+V)+J53 vs. (NHS+V)+J53+IR	0.0008
(iNHS+V)+J53 vs. (J53+V)+NHS	0.0008
(iNHS+V)+J53 vs. (J53+V)+iNHS	<0.0001
(iNHS+V)+J53 vs. (NHS+J53)+V	0.0008
(NHS+V)+J53+IR vs. (J53+V)+iNHS	<0.0001
(iNHS+V)+J53+IR vs. (J53+V)+iNHS	<0.0001
(J53+V)+NHS vs. (J53+V)+iNHS	<0.0001
(J53+V)+iNHS vs. (NHS+J53)+V	<0.0001
(J53+V)+iNHS vs. (iNHS+J53)+V	<0.0001

## Appendix D

**Table A3:** Mixed-model ANOVA results for each type of bacterial survival experiment divided by bacterial strains.

Experiments	Fixed Effects (Type III)	*p* Value	F (DFn, DFd)
bacteria+ NHS/iNHS	Time	0.2859	F (1.649, 6.594) = 1.491
	NHS activity	0.0257	F (1, 4) = 12.00
	Time × NHS activity	<0.0001	F (3, 12) = 17.98
bacteria+ nanocomposite	Time	0.0011	F (2.001, 16.01) = 10.75
	Nanocomposite	0.0012	F (3, 8) = 14.86
	Time × Nanocomposite	0.0014	F (9, 24) = 4.556
bacteria+ NHS+ nanocomposite	Time	<0.0001	F (1.865, 16.16) = 24.99
	Nanocomposite	0.076	F (3, 9) = 3.210
	Time × Nanocomposite	0.0724	F (9, 26) = 2.062
bacteria+ nanocomposite+ IR	Time	0.0028	F (1.319, 10.55) = 13.03
	Nanocomposite	0.0003	F (3, 8) = 22.32
	Time × Nanocomposite	0.0005	F (9, 24) = 5.372
(NHS+ nanocomposite)+ bacteria	Time	0.3299	F (1.231, 13.13) = 1.097
	Nanocomposite	<0.0001	F (3, 32) = 12.50
	Time × Nanocomposite	0.0501	F (9, 32) = 2.188
(iNHS+ nanocomposite)+ bacteria	Time	0.083	F (2.000, 18.00) = 2.866
	Nanocomposite	0.5903	F (3, 9) = 0.6723
	Time × Nanocomposite	0.0059	F (9, 27) = 3.458
(NHS+ nanocomposite)+ bacteria+ IR	Time	0.6662	F (2.311, 15.41) = 0.4609
	Nanocomposite	0.0028	F (3, 7) = 13.40
	Time × Nanocomposite	0.0007	F (9, 20) = 5.543
(iNHS+ nanocomposite)+ bacteria+ IR	Time	0.0074	F (1.498, 10.49) = 9.169
	Nanocomposite	0.0042	F (3, 7) = 11.61
	Time × Nanocomposite	0.013	F (9, 21) = 3.224
(bacteria+ nanocomposite)+ NHS	Time	<0.0001	F (1.520, 7.600) = 218.3
	Nanocomposite	0.0215	F (2, 5) = 9.123
	Time × Nanocomposite	0.0014	F (6, 15) = 6.644
(bacteria+ nanocomposite)+ iNHS	Time	0.2459	F (1.460, 7.301) = 1.677
	Nanocomposite	0.071	F (2, 5) = 4.701
	Time × Nanocomposite	0.0316	F (6, 15) = 3.197
(NHS+ bacteria)+ nanocomposite	Time	0.0033	F (1.533, 16.36) = 9.414
	Nanocomposite	<0.0001	F (3, 32) = 21.17
	Time × Nanocomposite	0.0161	F (9, 32) = 2.772
(iNHS+ bacteria)+ nanocomposite	Time	0.1946	F (2.089, 16.71) = 1.803
	Nanocomposite	0.0943	F (3, 8) = 3.013
	Time × Nanocomposite	0.1167	F (9, 24) = 1.819
All experiments with GO	Time	0.3581	F (1.791, 25.67) = 1.048
	Assay type	0.0008	F (8, 15) = 6.686
	Time × Assay type	0.0158	F (24, 43) = 2.115
All experiments with nanocomposite IV: GO-PcZr(Lys)2-Ag	Time	<0.0001	F (2.547, 63.66) = 9.845
	Assay type	0.0147	F (10, 25) = 2.910
	Time × Assay type	<0.0001	F (30, 75) = 3.616
All experiments with nanocomposite V: GO-Ag	Time	<0.0001	F (2.398, 52.75) = 20.64
	Assay type	<0.0001	F (10, 23) = 27.47
	Time × Assay type	<0.0001	F (30, 66) = 15.30
All experiments with nanocomposite VI: GO-PcZr(Lys)2	Time	0.6084	F (2.108, 40.06) = 0.5192
	Assay type	0.005	F (10, 19) = 3.939
	Time × Assay type	0.0102	F (30, 57) = 2.042
bacteria+ NHS/iNHS	Time	0.0168	F (1.367, 5.468) = 10.51
	NHS activity	0.0072	F (1, 4) = 25.52
	Time × NHS activity	<0.0001	F (3, 12) = 44.22
bacteria+ nanocomposite	Time	0.0319	F (1.965, 15.72) = 4.349
	Nanocomposite	0.0003	F (3, 8) = 22.21
	Time × Nanocomposite	<0.0001	F (9, 24) = 11.60
bacteria+ NHS+ nanocomposite	Time	<0.0001	F (1.457, 16.02) = 83.74
	Nanocomposite	0.0015	F (3, 11) = 10.46
	Time × Nanocomposite	<0.0001	F (9, 33) = 13.92
bacteria+ nanocomposite+ IR	Time	0.6116	F (1.678, 13.43) = 0.4535
	Nanocomposite	<0.0001	F (3, 8) = 74.37
	Time × Nanocomposite	<0.0001	F (9, 24) = 18.47
(NHS+ nanocomposite)+ bacteria	Time	<0.0001	F (1.460, 14.11) = 263.7
	Nanocomposite	0.0449	F (3, 10) = 3.872
	Time × Nanocomposite	<0.0001	F (9, 29) = 14.72
(iNHS+ nanocomposite)+ bacteria	Time	0.0921	F (1.881, 20.06) = 2.729
	Nanocomposite	0.009	F (3, 11) = 6.420
	Time × Nanocomposite	<0.0001	F (9, 32) = 11.52
(NHS+ nanocomposite)+ bacteria+ IR	Time	0.0001	F (1.020, 8.162) = 48.04
	Nanocomposite	0.7289	F (3, 8) = 0.4427
	Time × Nanocomposite	0.8765	F (9, 24) = 0.4758
(iNHS+ nanocomposite)+ bacteria+ IR	Time	0.1098	F (1.608, 17.69) = 2.614
	Nanocomposite	0.0059	F (3, 11) = 7.242
	Time × Nanocomposite	0.0518	F (9, 33) = 2.161
(bacteria+ nanocomposite)+ NHS	Time	<0.0001	F (1.008, 6.049) = 1725
	Nanocomposite	<0.0001	F (3, 6) = 69.34
	Time × Nanocomposite	<0.0001	F (9, 18) = 67.55
(bacteria+ nanocomposite)+ iNHS	Time	<0.0001	F (1.396, 7.909) = 61.74
	Nanocomposite	<0.0001	F (3, 6) = 127.6
	Time × Nanocomposite	<0.0001	F (9, 17) = 51.46
(NHS+ bacteria)+ nanocomposite	NA		
(iNHS+ bacteria)+ nanocomposite	Time	0.0093	F (1.957, 13.05) = 6.904
	Nanocomposite	0.1302	F (3, 7) = 2.650
	Time × Nanocomposite	0.0292	F (9, 20) = 2.736
All experiments with GO	Time	0.0767	F (1.560, 16.64) = 3.198
	Assay type	0.0046	F (9, 11) = 5.667
	Time × Assay type	0.0095	F (27, 32) = 2.393
All experiments with nanocomposite IV: GO-PcZr(Lys)2-Ag	Time	0.0093	F (1.936, 54.20) = 5.188
	Assay type	<0.0001	F (9, 28) = 15.78
	Time × Assay type	<0.0001	F (27, 84) = 6.024
All experiments with nanocomposite V: GO-Ag	Time	0.0542	F (2.134, 61.18) = 2.994
	Assay type	<0.0001	F (10, 29) = 244.4
	Time × Assay type	<0.0001	F (30, 86) = 88.45
All experiments with nanocomposite VI: GO-PcZr(Lys)2	Time	0.0112	F (1.310, 25.32) = 6.566
	Assay type	<0.0001	F (9, 20) = 8.579
	Time × Assay type	<0.0001	F (27, 58) = 4.693

## Appendix E

**Table A4:** Probability values of belonging to each cluster obtained by DTW/fuzzy clustering algorithms applied to all *E. coli* 6.2E survival time series. Cluster names were assigned based on their graphical representation (as illustrated in Figure A13).

Replicate	Weak Growth/Decline	Rapid Decline	Slight Decline	Growth	Moderate Decline
6.2E+NHS	0.393	0.168	0.252	0.095	0.092
6.2E+NHS_2	0.009	0.018	0.943	0.004	0.026
6.2E+NHS_3	0.422	0.215	0.224	0.068	0.071
6.2E+iNHS	0.154	0.033	0.075	0.708	0.031
6.2E+iNHS_2	0.179	0.111	0.189	0.400	0.121
6.2E+iNHS_3	0.183	0.102	0.187	0.408	0.120
6.2E+GO	0.042	0.701	0.147	0.013	0.097
6.2E+GO_2	0.042	0.701	0.147	0.013	0.097
6.2E+GO_3	0.042	0.701	0.147	0.013	0.097
6.2E+IV	0.240	0.078	0.338	0.033	0.311
6.2E+IV_2	0.201	0.081	0.519	0.073	0.126
6.2E+IV_3	0.362	0.114	0.282	0.047	0.196
6.2E+V	0.181	0.077	0.465	0.035	0.242
6.2E+V_2	0.325	0.113	0.313	0.064	0.185
6.2E+V_3	0.163	0.055	0.174	0.554	0.055
6.2E+VI	0.224	0.090	0.183	0.402	0.101
6.2E+VI_2	0.275	0.080	0.133	0.436	0.076
6.2E+VI_3	0.253	0.079	0.350	0.198	0.120
6.2E+NHS+GO	0.225	0.031	0.064	0.652	0.029
6.2E+NHS+GO_2	0.314	0.100	0.271	0.101	0.215
6.2E+NHS+GO_3	0.575	0.052	0.199	0.081	0.092
6.2E+NHS+IV	0.040	0.051	0.783	0.011	0.115
6.2E+NHS+IV_2	0.024	0.037	0.863	0.008	0.069
6.2E+NHS+IV_3	0.048	0.169	0.378	0.015	0.390
6.2E+NHS+V	0.113	0.214	0.320	0.012	0.341
6.2E+NHS+V_2	0.082	0.143	0.544	0.015	0.217
6.2E+NHS+V_3	0.435	0.188	0.236	0.067	0.075
6.2E+NHS+V_4	0.336	0.067	0.239	0.033	0.325
6.2E+NHS+VI	0.076	0.099	0.124	0.008	0.693
6.2E+NHS+VI_2	0.273	0.168	0.200	0.036	0.323
6.2E+NHS+VI_3	0.019	0.299	0.057	0.004	0.621
6.2E+GO+IR	0.042	0.701	0.147	0.013	0.097
6.2E+GO+IR_2	0.042	0.701	0.147	0.013	0.097
6.2E+GO+IR_3	0.042	0.701	0.147	0.013	0.097
6.2E+IV+IR	0.000	0.000	1.000	0.000	0.000
6.2E+IV+IR_2	0.131	0.191	0.490	0.047	0.141
6.2E+IV+IR_3	0.104	0.125	0.665	0.031	0.076
6.2E+V+IR	0.590	0.036	0.099	0.231	0.044
6.2E+V+IR_2	0.224	0.068	0.277	0.026	0.405
6.2E+V+IR_3	0.172	0.139	0.433	0.036	0.220
6.2E+VI+IR	0.518	0.071	0.126	0.187	0.097
6.2E+VI+IR_2	0.173	0.069	0.155	0.511	0.091
6.2E+VI+IR_3	0.218	0.132	0.176	0.309	0.165
(NHS+GO)+6.2E	0.261	0.050	0.095	0.546	0.047
(NHS+GO)+6.2E_2	0.262	0.119	0.168	0.336	0.115
(NHS+GO)+6.2E_3	0.294	0.132	0.198	0.235	0.141
(NHS+IV)+6.2E	0.329	0.039	0.110	0.467	0.056
(NHS+IV)+6.2E_2	0.008	0.001	0.003	0.987	0.001
(NHS+IV)+6.2E_3	0.061	0.010	0.031	0.888	0.009
(NHS+V)+6.2E	0.000	1.000	0.000	0.000	0.000
(NHS+V)+6.2E_2	0.095	0.080	0.625	0.025	0.175
(NHS+V)+6.2E_3	0.364	0.183	0.226	0.090	0.137
(NHS+VI)+6.2E	0.026	0.388	0.518	0.007	0.060
(NHS+VI)+6.2E_2	0.331	0.127	0.283	0.024	0.236
(NHS+VI)+6.2E_3	0.049	0.389	0.404	0.012	0.146
(iNHS+GO)+6.2E	0.512	0.082	0.177	0.106	0.124
(iNHS+GO)+6.2E_2	0.258	0.138	0.183	0.288	0.134
(iNHS+IV)+6.2E	0.291	0.041	0.109	0.517	0.042
(iNHS+IV)+6.2E_2	0.275	0.086	0.387	0.143	0.109
(iNHS+IV)+6.2E_3	0.398	0.076	0.161	0.277	0.087
(iNHS+IV)+6.2E_4	0.275	0.087	0.141	0.414	0.083
(iNHS+IV)+6.2E_5	0.385	0.024	0.079	0.486	0.027
(iNHS+IV)+6.2E_6	0.541	0.064	0.107	0.039	0.249
(iNHS+V)+6.2E	1.000	0.000	0.000	0.000	0.000
(iNHS+V)+6.2E_2	0.456	0.172	0.222	0.066	0.084
(iNHS+V)+6.2E_3	0.379	0.166	0.254	0.105	0.096
(iNHS+VI)+6.2E	0.273	0.078	0.255	0.200	0.194
(iNHS+VI)+6.2E_2	0.238	0.111	0.166	0.378	0.107
(NHS+GO)+6.2E+IR	0.264	0.115	0.165	0.345	0.111
(NHS+GO)+6.2E+IR_2	0.273	0.094	0.148	0.395	0.090
(NHS+IV)+6.2E+IR	0.127	0.132	0.486	0.041	0.215
(NHS+IV)+6.2E+IR_2	0.449	0.100	0.174	0.128	0.149
(NHS+IV)+6.2E+IR_3	0.297	0.086	0.189	0.027	0.402
(NHS+V)+6.2E+IR	0.290	0.100	0.116	0.018	0.477
(NHS+V)+6.2E+IR_2	0.480	0.128	0.262	0.063	0.066
(NHS+V)+6.2E+IR_3	0.338	0.063	0.179	0.319	0.102
(NHS+VI)+6.2E+IR	0.226	0.341	0.254	0.025	0.153
(NHS+VI)+6.2E+IR_2	0.056	0.188	0.704	0.021	0.031
(NHS+VI)+6.2E+IR_3	0.036	0.549	0.234	0.007	0.174
(iNHS+GO)+6.2E+IR	0.248	0.243	0.215	0.124	0.170
(iNHS+GO)+6.2E+IR_2	0.352	0.066	0.138	0.377	0.067
(iNHS+IV)+6.2E+IR	0.185	0.066	0.129	0.558	0.062
(iNHS+IV)+6.2E+IR_2	0.230	0.044	0.087	0.598	0.041
(iNHS+IV)+6.2E+IR_3	0.230	0.032	0.067	0.641	0.030
(iNHS+V)+6.2E+IR	0.260	0.123	0.171	0.328	0.119
(iNHS+V)+6.2E+IR_2	0.252	0.135	0.179	0.303	0.131
(iNHS+V)+6.2E+IR_3	0.243	0.148	0.185	0.280	0.144
(iNHS+VI)+6.2E+IR	0.274	0.090	0.143	0.408	0.085
(iNHS+VI)+6.2E+IR_2	0.189	0.128	0.220	0.297	0.166
(iNHS+VI)+6.2E+IR_3	0.215	0.024	0.069	0.669	0.023
(6.2E+GO)+NHS	0.000	1.000	0.000	0.000	0.000
(6.2E+IV)+NHS	0.034	0.185	0.265	0.010	0.506
(6.2E+IV)+NHS_2	0.016	0.768	0.175	0.005	0.036
(6.2E+IV)+NHS_3	0.066	0.168	0.281	0.015	0.470
(6.2E+V)+NHS	0.021	0.602	0.060	0.005	0.312
(6.2E+V)+NHS_2	0.021	0.298	0.065	0.005	0.611
(6.2E+V)+NHS_3	0.016	0.791	0.055	0.004	0.134
(6.2E+VI)+NHS	0.020	0.785	0.101	0.005	0.089
(6.2E+VI)+NHS_2	0.021	0.822	0.089	0.006	0.063
(6.2E+GO)+iNHS	0.000	1.000	0.000	0.000	0.000
(6.2E+IV)+iNHS	0.000	0.000	0.000	0.000	1.000
(6.2E+IV)+iNHS_2	0.262	0.078	0.131	0.449	0.080
(6.2E+IV)+iNHS_3	0.275	0.083	0.136	0.426	0.079
(6.2E+V)+iNHS	0.021	0.602	0.060	0.005	0.312
(6.2E+V)+iNHS_2	0.021	0.298	0.065	0.005	0.611
(6.2E+V)+iNHS_3	0.043	0.380	0.048	0.007	0.522
(6.2E+VI)+iNHS	0.185	0.051	0.132	0.557	0.076
(6.2E+VI)+iNHS_2	0.100	0.020	0.052	0.807	0.020
(NHS+6.2E)+GO	0.000	0.000	0.000	1.000	0.000
(NHS+6.2E)+GO_2	0.281	0.115	0.219	0.253	0.132
(NHS+6.2E)+GO_3	0.153	0.026	0.085	0.713	0.023
(NHS+6.2E)+IV	0.150	0.126	0.547	0.036	0.141
(NHS+6.2E)+IV_2	0.299	0.111	0.332	0.109	0.149
(NHS+6.2E)+IV_3	0.037	0.007	0.017	0.930	0.007
(NHS+6.2E)+V	0.045	0.621	0.202	0.013	0.119
(NHS+6.2E)+V_2	0.042	0.678	0.165	0.012	0.103
(NHS+6.2E)+V_3	0.041	0.685	0.160	0.013	0.101
(NHS+6.2E)+VI	0.085	0.067	0.765	0.020	0.064
(NHS+6.2E)+VI_2	0.364	0.144	0.169	0.107	0.217
(NHS+6.2E)+VI_3	0.083	0.148	0.433	0.012	0.323
(iNHS+6.2E)+GO	0.291	0.104	0.357	0.091	0.156
(iNHS+6.2E)+GO_2	0.324	0.077	0.188	0.337	0.075
(iNHS+6.2E)+GO_3	0.297	0.108	0.182	0.303	0.110
(iNHS+6.2E)+IV	0.416	0.085	0.191	0.063	0.244
(iNHS+6.2E)+IV_2	0.266	0.078	0.125	0.458	0.074
(iNHS+6.2E)+IV_3	0.254	0.062	0.110	0.511	0.063
(iNHS+6.2E)+V	0.254	0.044	0.086	0.575	0.041
(iNHS+6.2E)+V_2	0.221	0.135	0.346	0.090	0.208
(iNHS+6.2E)+V_3	0.257	0.056	0.107	0.492	0.088
(iNHS+6.2E)+VI	0.221	0.176	0.196	0.234	0.174
(iNHS+6.2E)+VI_2	0.245	0.145	0.184	0.285	0.141
(iNHS+6.2E)+VI_3	0.222	0.099	0.155	0.426	0.099
